# Effect of Antimicrobial Prophylaxis Duration on Health Care–Associated Infections After Clean Orthopedic Surgery: A Cluster Randomized Trial

**DOI:** 10.1001/jamanetworkopen.2022.6095

**Published:** 2022-04-12

**Authors:** Kosei Nagata, Koji Yamada, Tomohiro Shinozaki, Tsuyoshi Miyazaki, Fumiaki Tokimura, Yasuhito Tajiri, Takuya Matsumoto, Kiyofumi Yamakawa, Hiroyuki Oka, Akiro Higashikawa, Toshihide Sato, Kenichi Kawano, Tatsuro Karita, Takuya Koyama, Takahiro Hozumi, Hiroaki Abe, Makoto Hodohara, Kazuhiro Kohata, Masato Toyonaga, Yasushi Oshima, Sakae Tanaka, Hiroshi Okazaki

**Affiliations:** 1 Department of Orthopedic Surgery and Spinal Surgery, the University of Tokyo Hospital, Tokyo, Japan; 2 Department of Orthopedic Surgery, Japan Organization of Occupational Health and Safety Kanto Rosai Hospital, Kanagawa, Japan; 3 Department of Information and Computer Technology, Faculty of Engineering, Tokyo University of Science, Tokyo, Japan; 4 Department of Orthopedic Surgery, Tokyo Metropolitan Geriatric Hospital and Institute of Gerontology, Tokyo, Japan; 5 Department of Orthopedic Surgery, Tokyo Metropolitan Hiroo Hospital, Tokyo, Japan; 6 Department of Orthopedic Surgery, Tokyo Metropolitan Tama Medical Center, Tokyo, Japan; 7 Department of Orthopedic Surgery, Tokyo Metropolitan Cancer and Infectious Diseases Center Komagome Hospital, Tokyo, Japan; 8 Department of Medical Research and Management for Musculoskeletal Pain, 22nd Century Medical and Research Center, Faculty of Medicine, The University of Tokyo, Tokyo, Bunkyo-ku, Japan

## Abstract

**Importance:**

Postoperative health care–associated infections are associated with a greater deterioration in patients’ general health status and social and economic burden, with at least 1 occurring in approximately 4% of acute care hospital patients. Antimicrobial prophylaxis prevents surgical site infections in various orthopedic procedures; however, its relationship with health care–associated infections remains unknown.

**Objective:**

To examine whether a shorter antimicrobial prophylaxis duration of less than 24 hours after surgery is not inferior to a longer duration in preventing health care–associated infections after clean orthopedic surgery.

**Design, Setting, and Participants:**

This open-label, multicenter, cluster randomized, noninferiority clinical trial was conducted in 5 tertiary referral hospitals in greater Tokyo metropolitan area, Japan, from May to December 2018. Adult patients undergoing clean orthopedic surgery were recruited until the planned number of participants was achieved (500 participants per group). Statistical analysis was conducted from July to December 2019.

**Interventions:**

Antimicrobial prophylaxis was discontinued within 24 hours after surgery in group 24 and 24 to 48 hours after surgery in group 48. Group allocation was switched every 2 or 4 months according to the facility-based cluster rule. Study-group assignments were masked from participants.

**Main Outcomes and Measures:**

The primary outcome was the incidence of health care–associated infections requiring antibiotic therapies within 30 days after surgery. The noninferiority margin was 4%.

**Results:**

Of the 1211 participants who underwent cluster allocation, 633 participants were in group 24 (median [IQR] age, 73 [61-80] years; 250 men [39.5%] and 383 women [60.5%]), 578 participants were in group 48 (median [IQR] age, 74 [62-81] years; 204 men [35.3%] and 374 women [64.7%]), and all were eligible for the intention-to-treat analyses. Health care–associated infections occurred in 29 patients (4.6%) in group 24 and 38 patients (6.6%) in group 48. Intention-to-treat analyses showed a risk difference of −1.99 percentage points (95% CI, −5.05 to 1.06 percentage points; *P* < .001 for noninferiority) between groups, indicating noninferiority. Results of adjusted intention-to-treat, per-protocol, and per designated procedure population analyses supported this result, without a risk of antibiotic resistance and prolonged hospitalization.

**Conclusions and Relevance:**

This cluster randomized trial found noninferiority of a shorter antimicrobial prophylaxis duration in preventing health care–associated infections without an increase in antibiotic resistance risk. These findings lend support to the global movement against antimicrobial resistance and provide additional information on adequate antimicrobial prophylaxis for clean orthopedic surgery.

**Trial Registration:**

Identifier: UMIN000030929

## Key Points

**Question:**

Is a shorter antimicrobial prophylaxis duration (<24 hours after surgery) inferior to a longer duration (24-48 hours) in preventing health care–associated infections after clean orthopedic surgery?

**Findings:**

In this cluster randomized clinical trial of 1211 adults who underwent clean orthopedic surgery, health care–associated infections occurred in 4.6% of patients in the group with shorter duration and 6.6% of patients in the group with longer duration, indicating noninferiority of the shorter antimicrobial prophylaxis duration without significant difference in prolonged hospitalization, antibiotic resistance, and serious adverse events.

**Meaning:**

These findings suggest support for the use of a shorter antimicrobial prophylaxis duration and a lower antibiotic load in clean orthopedic surgery.

## Teaser

This cluster randomized trial investigates whether a shorter antimicrobial prophylaxis (AMP) duration of less than 24 hours after surgery is not inferior to a longer duration in preventing health care–associated infections after clean orthopedic surgery.

## Introduction

Postoperative health care–associated infections (HAIs), including surgical site infections (SSIs), urinary tract infections (UTIs), and respiratory tract infections (RTIs), are associated with greater deterioration in patients’ general health status and social and economic burden.^1,2^ Approximately 4% of the patients in acute care hospitals in the United States have at least 1 HAI.^1^ More than half of HAIs are considered preventable by an evidence-based approach,^2^ and perioperative antibiotics administration may play an important role in infection prevention.^3,4^

Antimicrobial prophylaxis (AMP) is one of the most important methods for preventing SSIs.^3,5,6^ The World Health Organization (WHO)^5^ and the US Center for Disease Control and Prevention (CDC)^3^ guidelines recently recommended AMP without additional doses after wound closure. However, this recommendation has been challenged by several societies because of insufficient evidence.^6,7,8,9,10^ These societies recommended the discontinuation of AMP within 24 hours after clean orthopedic surgeries.^7,9^ Some recommended an even longer duration (within 48 hours) after spinal instrumentation and arthroplasties surgeries.^6,10^ However, infectious disease specialists have criticized the prolongation of AMP duration, as it may increase the risk of antimicrobial resistance^4^ and other hazardous side effects.^11,12^ Our recent study showed that about half of the orthopedic surgeons still prescribe AMP for more than 24 hours after wound closure.^13^ Several multicountry studies also found that AMP is still routinely continued for several days after surgery.^14,15^ Therefore, additional evidence is needed for orthopedic surgeons to minimize the AMP duration.

Although the primary aim of AMP is to prevent SSIs, AMP duration may have additional benefits in preventing other HAIs. However, its clinical effect has not been well investigated. In this study, we aimed to examine the noninferiority of a shorter AMP duration (within 24 hours after surgery) against a longer duration (within 48 hours) in preventing HAIs after clean orthopedic surgery.

## Methods

### Design Overview

The Non-inferior Comparative Study Comparing One- or Two-day Antimicrobial Prophylaxis after Clean Orthopedic Surgery (NOCOTA study) was a multicenter, open-label, cluster randomized, clinical trial conducted by the Society of Orthopedic Surgical Site Infection (OSSI) investigators.^13,16^ The study protocol was approved by the institutional ethics boards of the 5 participating hospitals: Tokyo Metropolitan Tama Medical Center, Tokyo Metropolitan Hiroo Hospital, Tokyo Metropolitan Geriatric Hospital and Institute of Gerontology, Tokyo Metropolitan Cancer and Infectious Diseases Center Komagome Hospital, and Japan Organization of Occupational Health and Safety Kanto Rosai Hospital. The methodological details of the trial have been published elsewhere (Supplement 1).^16^ All participants provided written informed consent before the study. We followed the Consolidated Standards of Reporting Trials (CONSORT) reporting guideline for cluster randomized clinical trials.

### Patient Population

Eligible participants were aged at least 20 years and included patients who were hospitalized, underwent clean surgery based on the CDC wound classification system, had a good command of the Japanese language, could give consent on their own or through relatives, and had a primary intention wound closure. Exclusion criteria were operations involving the use of external fixations, amputations, needle biopsies, implant removal, reconstructive surgery involving skin tissue such as flap surgery, antibiotic therapy before surgery, percutaneous vertebroplasty, and procedures performed together with other departments, according to our previous study.^13^ For this trial, we also excluded patients taking antibiotic, antiviral, antifungal, or antituberculosis drugs on the day of surgery; those who underwent operations deemed inappropriate for study continuation owing to adverse events and deterioration of complications or of the original disease; any exclusion criterion applicable after registration; patients who were discharged within 48 hours after surgery; patients who refused to participate; and inappropriate continuation of antibiotics for other rational reasons.

### Randomization and Intervention

Cluster randomization was performed using the institution-by-period cluster allocation system. Patients were divided into 2 groups depending on AMP duration. AMP administration after wound closure was discontinued within 24 hours in group 24 and within 24 to 48 hours in group 48. AMP duration was defined as the time from when the wound was closed to the time when the final antibiotic was administered. The intervention was switched every 2 or 4 months in each institution until the planned number of participants was achieved.^16^

The allocation schedule was fixed by OSSI investigators^13,16^ and announced to participating physicians before the study. Each hospital had a coordinator, and each hospital representative was responsible for data validation per-protocol. Participating hospitals were asked not to select patients based on AMP duration to minimize selection bias and prohibit crossovers. At recruitment, we asked all patients to provide their contact number and to report all postoperative infectious complications.

Study-group assignments were masked from participants. However, participants might have noticed their allocated assignments when they received additional antibiotics 24 hours after the surgery.

### Procedure

All patients followed the domestic guideline^6^ and received an initial dose of 2 g of cefazolin before incision, with an additional 1 g provided every 3 to 4 hours during surgery. Vancomycin or clindamycin was used for patients with beta-lactam allergy. Preoperative AMP administration was considered adequate if cefazolin was started within 60 minutes before the surgical incision (120 minutes for vancomycin). Additional doses during surgery were provided based on renal function following the domestic guidelines.^6,10^ Participating physicians were asked to report all bacterial infectious diseases and obtain cultures to detect the source and causative pathogen. When infectious diseases were suspected, the participating physicians were asked to provide appropriate therapy. No restriction was set on antimicrobial choice or duration for the purpose of treatment. To minimize the effect of confounding factors, all participating institutions agreed to follow the SSI prevention protocols described in the protocol paper^16^ (summarized in eTable 1 in Supplement 2).

### Outcome Measures

The primary outcome was HAIs of all postoperative bacterial infectious diseases diagnosed within 30 days after surgery requiring antibiotic therapy. The operation date was defined as day 0. HAIs were classified into SSIs, UTIs, RTIs, or other infectious diseases diagnosed based on the modified CDC definitions, as described previously.^13^ Secondary outcomes included the prevalence of (1) SSIs, (2) UTIs, (3) RTIs, (4) other infectious diseases, (5) life-threatening cardiovascular events, and (6) mortality observed within 30 days after surgery; (7) days of hospitalization; and (8) the rate of antibiotic resistance observed among SSI and HAI pathogens. All outcomes were dichotomized at specific follow-up dates.

The outcomes were evaluated within 30 to 180 days after the surgery directly by physicians or via telephone or postal postdischarge surveys. Adverse events, including anaphylaxis of AMP or pseudomembranous colitis, were also assessed.

### Statistical Analysis

The target sample size of 500 participants per group was based on the Farrington-Manning noninferiority score test, setting a 4% anticipated rate of HAIs within 30 days after surgery (in reference to our previous study),^13^ a 4-percentage-point noninferiority margin on the absolute risk difference scale (corresponding to a relative difference of 100%), with a 1-sided α level of 0.025, 80% power, and 20% loss to follow-up. Participant recruitment started on May 2018, steadily increased throughout our study period (eFigure 1 in Supplement 2), and was completed in December 2018 (at the end of the fourth term).

Both intention-to-treat (ITT) and per-protocol analyses were performed as primary analyses. ITT analysis comparing the groups were defined according to the allocation. The risks of the primary outcome were compared between the groups. We also estimated the crude and multivariable-adjusted risk differences and ratios with their 95% CIs using linear and log-linear regression models with canonical links fitted by generalized estimating equations (GEEs) that cluster hospitals and periods. In addition to analysis of the ITT population, we performed analysis using the per-protocol population, excluding patients who deviated from the allocation. We also performed further analysis using a per designated procedure population, excluding those who used local antibiotics and those who did not use iodine-impregnated adhesive drapes or antimicrobial-coated sutures for SSI prevention. We plotted the cumulative incidence curves of HAIs and SSIs over 30 days after the surgery and compared the hazards of the 2 groups using Cox models with cluster-robust standard errors.

For secondary outcomes, we evaluated whether the corresponding risks in group 24 were excessively higher than those in group 48 by estimating the 95% CI of risk differences and ratios using GEE analyses. We further performed subgroup analyses of HAIs and SSIs by the patients’ background and surgical factors.

Statistical analyses were performed using SAS version 9.4 (SAS Institute) from July to December 2019. A significance threshold was set at *P* < .025 for the 1-sided Farrington–Manning test of the HAI risk difference of 4%.

## Results

### Patients

**Figure 1.**
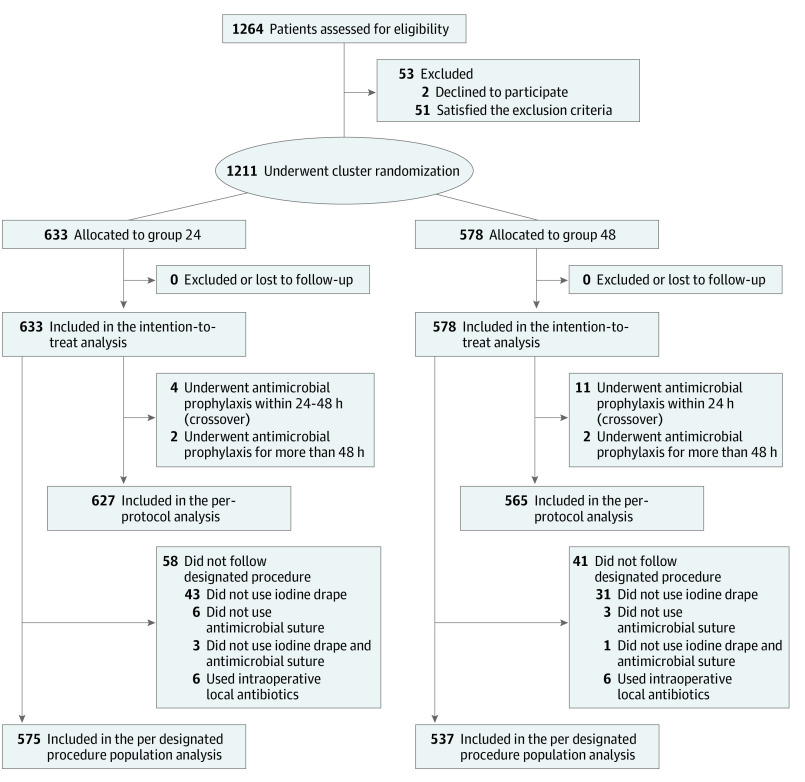
Diagram of Participant Flow Through Trial

In total, 1264 participants were assessed for eligibility. After excluding 53 participants who declined to participate or met the exclusion criteria, 1211 participants were allocated to either group 24 or group 48 (Figure 1). Using the cluster rule, we allocated 633 participants (median [IQR] age, 73 (61-80) years; 383 women [60.5%] and 250 men [39.5%]) to group 24 and 578 (median [IQR] age, 74 [62-81] years; 374 women [64.7%] and 204 men [35.3%] ) to group 48. All participants completed the 30-day follow-up within 30 to 180 days after surgery (eFigure 2 in Supplement 2); therefore, the ITT analysis included 1211 participants. The participants were well matched with regard to the baseline characteristics (Table 1). All patients received initial AMP, as appropriate. No adverse events associated with AMP, including anaphylaxis and pseudomembranous colitis, were reported. In total, 1192 participants were considered as the per-protocol population: 627 in group 24 and 565 in group 48 (Figure 1). After investigating the application of iodine-impregnated adhesive drapes, antimicrobial-coated sutures, and local antibiotics, we examined 1112 participants as the per-designated procedure population: 575 in group 24 and 537 in group 48 (Figure 1). More than 97% of the participants were evaluated in-person by surgeons in charge of the operating hospital or by external physicians in each group (Table 1). There were no postal surveys. Surgeons in charge conducted 2.4% and 2.1% of the telephone surveys in groups 24 and group 48, respectively.

**Table 1.** Baseline Characteristics of the Trial Participants

Characteristics	Participants, No. (%)	
	Group 24 (n = 633)	Group 48 (n = 578)
Age, median (IQR), y	73 (61-80)	74 (62-81)
Sex		
Female	383 (60.5)	374 (64.7)
Male	250 (39.5)	204 (35.3)
Body mass index, median (IQR)^a^	23.4 (20.6-26.1)	23.0 (20.9-26.2)
Smokers	69 (10.9)	62 (10.7)
Diabetes	72 (11.4)	59 (10.2)
Hemodialysis	5 (0.8)	4 (0.7)
Rheumatoid arthritis	27 (4.3)	32 (5.5)
American Society of Anesthesiologists physical status		
1	80 (12.6)	68 (11.8)
2	490 (77.4)	455 (78.7)
3	62 (9.8)	55 (9.5)
4	1 (0.2)	0
Operative time, mean (SD), min	126.2 (72.9)	133.7 (77.1)
Estimated blood loss, mean (SD), mL	234.3 (288.8)	254.2 (361.6)
Surgery type		
Open reduction internal fixation	119 (18.8)	117 (20.2)
Arthroplasty	273 (43.1)	245 (42.4)
Arthrodesis	6 (0.9)	3 (0.5)
Osteotomy	6 (0.9)	2 (0.3)
Spine without instrumentation	108 (17.1)	98 (17.0)
Spine with instrumentation	94 (14.8)	93 (16.1)
Arthroscopy	10 (1.6)	9 (1.6)
Bone tumor resection	0	1 (0.2)
Soft tissue tumor resection	3 (0.5)	3 (0.5)
Other	14 (2.2)	7 (1.2)
Type of anesthesia		
General	502 (79.3)	448 (77.5)
Spinal	130 (10.5)	134 (23.2)
Epidural	83 (13.1)	86 (14.9)
Peripheral block	29 (4.6)	23 (4.0)
Emergency surgery	21 (3.3)	22 (3.8)
Revision surgery	25 (3.9)	38 (6.6)
Antimicrobial prophylaxis		
Cefazolin	623 (98.4)	575 (99.5)
Vancomycin	8 (1.3)	4 (0.7)
Clindamycin	47 (7.4)	47 (8.1)
Nasal decolonization	40 (6.3)	54 (9.3)
Skin preparation		
Iodine-alcohol	400 (63.2)	343 (59.3)
Iodine	228 (36.0)	225 (38.9)
Chlorhexidine alcohol	4 (0.6)	9 (1.6)
0.5% Chlorhexidine gluconate	0	1 (0.2)
Olanexidine gluconate	1 (0.2)	0
Iodine drape use	587 (92.7)	546 (94.5)
Diluted iodine irrigation	187 (29.5)	204 (35.3)
Intraoperative local antibiotics use	9 (1.4)	4 (0.7)
Cement	3 (0.5)	0
Vancomycin powder	6 (0.9)	4 (0.7)
Antimicrobial suture	627 (99.1)	572 (99.0)
Body temperature at the end of procedure		
Normothermia (≥36 °C)	529 (83.6)	496 (85.8)
Hypothermia (<36 °C)	85 (13.4)	69 (11.9)
Unmeasured	19 (3.0)	13 (2.2)
Urinary catheter		
None	84 (13.3)	46 (8.0)
<24 h	159 (25.1)	175 (30.3)
≥24 h	390 (61.6)	357 (61.8)
Type of surgical drain, duration		
Closed		
<48 h	296 (46.8)	256 (44.3)
≥48 h	258 (40.8)	253 (43.8)
None	67 (10.6)	66 (11.4)
Not closed		
<48 h	12 (1.9)	3 (0.5)
≥48 h	0	0
Hospital		
1	310 (49.0)	263 (45.5)
2	111 (17.5)	79 (13.7)
3	56 (8.8)	56 (9.7)
4	48 (7.6)	66 (11.4)
5	108 (17.1)	114 (19.7)
Period		
May-June, 2018	219 (34.6)	157 (27.2)
July-August, 2018	138 (21.8)	189 (32.7)
Sept-Oct, 2018	137 (21.6)	140 (24.2)
Nov-Dec, 2018	139 (22.0)	92 (15.9)
Assessors/methods		
Surgeons-in-charge within the operating hospital/in-person evaluation	599 (94.6)	548 (94.8)
Physicians outside the operating hospital/in-person evaluation	19 (3.0)	18 (3.1)
Surgeons-in-charge within the operating hospital/postal survey	0	0
Surgeons-in-charge within the operating hospital/telephone survey	15 (2.4)	12 (2.1)

^a^ Body mass index is calculated as weight in kilograms divided by height in meters squared.

### Primary Outcome

**Figure 2.**
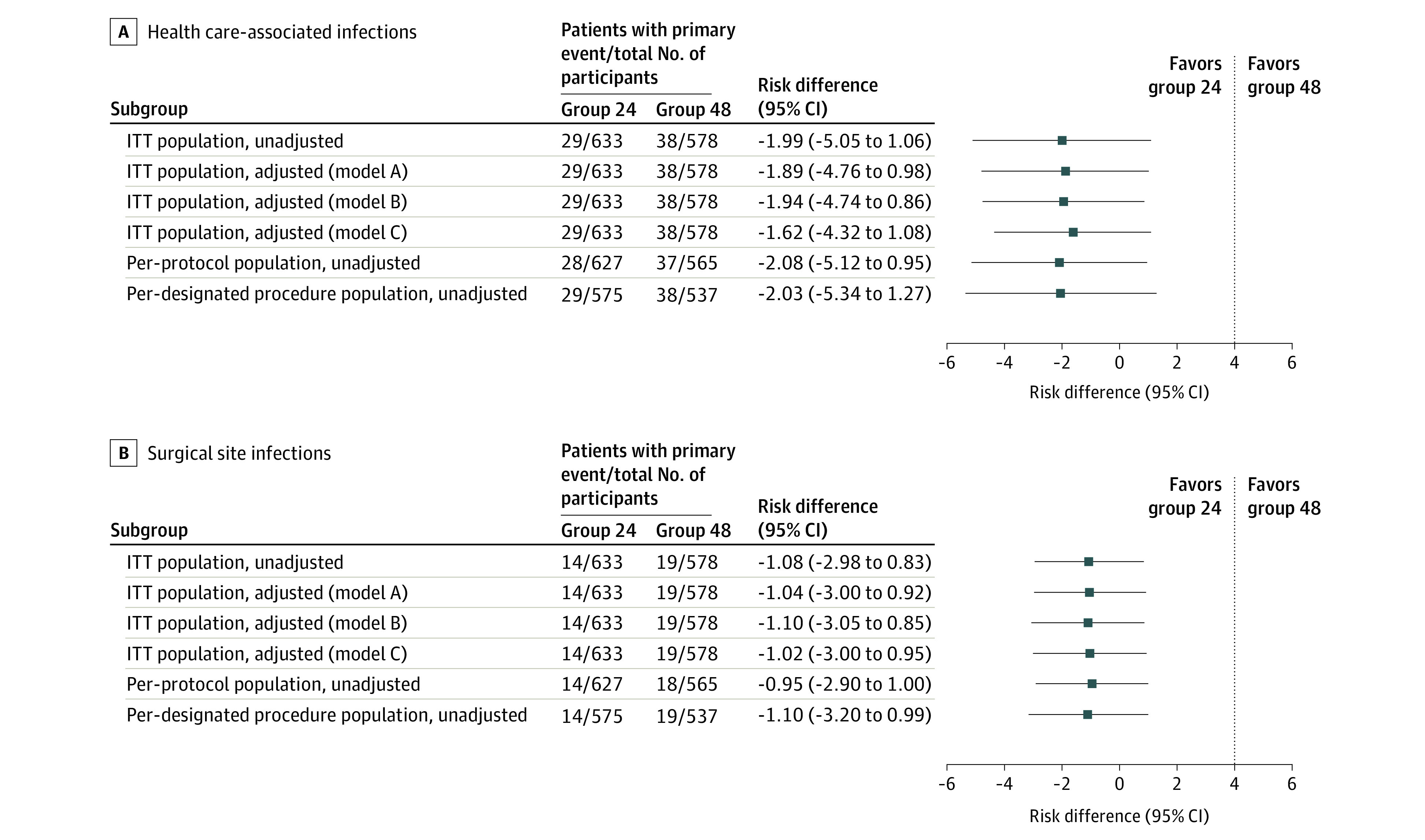
Differences in Risk According to the Analysis Performed The point estimates for the differences in the occurrence of health care–associated infections (HAIs) (panel A) and surgical site infections (panel B) are shown with 2-sided 95% CIs. The vertical dotted line indicates the noninferiority margin. Noninferiority was consistent in both the intention-to-treat (ITT) and per-protocol populations in terms of HAIs. For the ITT populations, model A was adjusted for operation type. For model B, in addition to the operation type, the following variables were included: age (<65 vs ≥65 years), sex, diabetes, American Society of Anesthesiologists score (grouping 1 and 2, and 3 and 4), body mass index (BMI, calculated as weight in kilograms divided by height in meters squared) (<30 vs ≥30), operative time (<150 min vs ≥150 min), and estimated blood loss (<1000 mL vs ≥1000 mL). For model C, general anesthesia (any vs none) and use of urinary catheter (any vs none) were also included.

**Figure 3.**
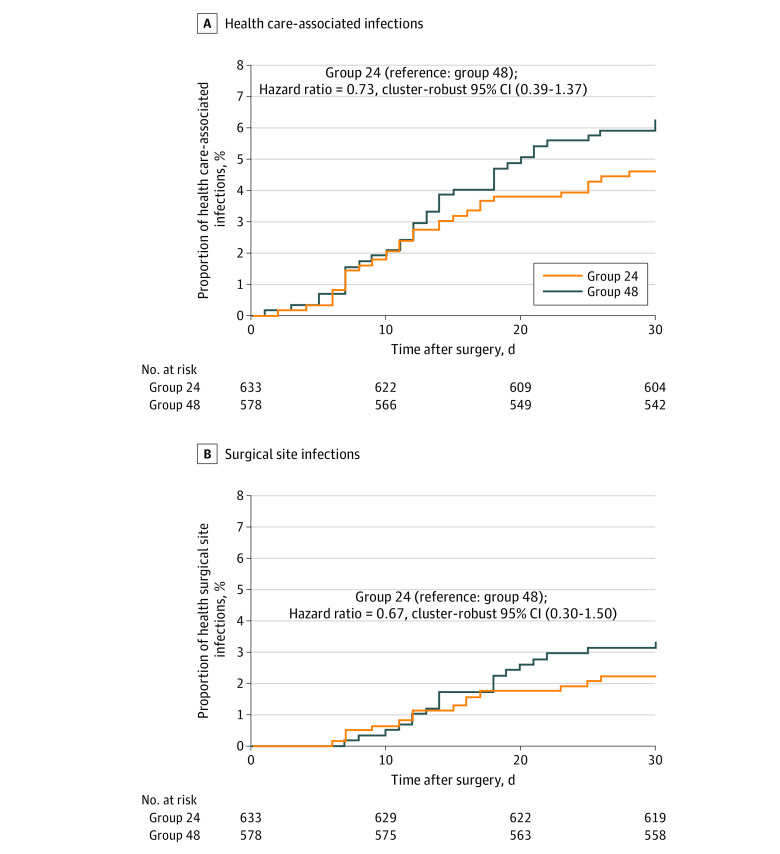
Cumulative Incidence Probability of Health Care–Associated Infections and Surgical Site Infections in Group 24 and Group 48 The hazard ratio of group 24 vs group 48 was estimated using the Cox proportional hazard model in the intention-to-treat population for health care–associated infections (HAIs) (panel A) and surgical site infections (SSIs) (panel B).

In the ITT population, 29 HAIs (4.6%) occurred in group 24 and 38 HAIs (6.6%) occurred in group 48 (Table 2). Detailed descriptions of HAIs are available in eTable 2 in Supplement 2. The difference in the risk of HAI of group 24 vs group 48 in the ITT population was −1.99 percentage points (95% CI, −5.05 to 1.06 percentage points; *P* < .001 for noninferiority); thus meeting the 4-percentage-point margin (Figure 2A). A similar trend was observed when omitting culture negative cases (eTable 3 in Supplement 2). In the per-protocol population, 28 HAIs (4.5%) were observed in group 24 and 37 HAIs (6.5%) were observed in groups 48, indicating that 1 HAI occurred in those who violated the study protocol in each group. The results of the adjusted ITT analyses, per-protocol analysis, and analysis in per designated procedure population were all consistent with those of the ITT analyses when the 4-percentage-point margin was used (Figure 2A). The hazard ratio (HR) for HAI within 30 days after surgery in group 24 vs group 48 was 0.73 (95% CI, 0.39 to 1.37) (Figure 3A). In subgroup analyses, no advantage of the longer AMP was observed (eFigure 3 in Supplement 2).

**Table 2.** Primary and Secondary End Points in the Intention-to-Treat Population^a^

End Point	Event, No. (%)		Risk difference, percentage points (95% CI), %^b^	Risk ratio, (95% CI), %^b^
	Group 24 (n = 633)	Group 48 (n = 578)		
Primary				
HAI	29 (4.6)	38 (6.6)	−1.99 (−5.05 to 1.06)	0.70 (0.38 to 1.27)
Secondary				
SSI	14 (2.2)	19 (3.3)	−1.08 (−2.98 to 0.83)	0.67 (0.30 to 1.49)
Deep	4 (0.6)	7 (1.2)	−1.74 (−4.34 to 0.87)	0.52 (0.18 to 1.52)
UTI	11 (1.7)	13 (2.2)	−0.51 (−2.53 to 1.51)	0.77 (0.29 to 2.05)
RTI	3 (0.5)	5 (0.9)	−0.39 (−1.41 to 0.63)	0.55 (0.11 to 2.63)
Other infectious diseases	3 (0.5)	2 (0.3)	0.13 (−0.34 to 0.60)	1.37 (0.41 to 4.60)
Death	0	2 (0.3)	−0.35 (−0.77 to 0.08)	NA
Life-threatening cardiovascular events	0	0	NA	NA
Readmission	3 (0.5)	4 (0.7)	−0.22 (−0.87 to 0.43)	0.68 (0.24 to 1.96)
Days of hospitalization				
7	569 (89.9)	532 (92.0)	−2.15 (−7.68 to 3.38)	0.98 (0.92 to 1.04)
14	430 (67.9)	413 (71.5)	−3.52 (−11.71 to 4.67)	0.95 (0.85 to 1.07)
21	202 (31.9)	214 (37.0)	−5.11 (−17.95 to 7.73)	0.86 (0.59 to 1.25)
30	75 (11.8)	89 (15.4)	−3.55 (−12.27 to 5.17)	0.77 (0.41 to 1.45)
Antibiotic resistance				
Of SSI pathogens^c^	0	2 (0.3)	−0.35 (−0.73 to 0.04)	NA
Of HAI pathogens^c^	2 (0.3)	4 (0.7)	−0.38 (−1.32 to 0.59)	0.46 (0.06 to 3.61)
Serious adverse events				
Anaphylaxis related to AMP	0	0	NA	NA
Pseudomembranous colitis	0	0	NA	NA

Abbreviations: AMP, antimicrobial prophylaxis; HAI, health care–associated infection; NA, not applicable; RTI, respiratory tract infection; SSI, surgical site infection; UTI, urinary tract infection.

^a^ All outcomes were dichotomized into presence or absence at patient-level.

^b^ Estimated by linear- and log-linear-risk generalized estimating equations (with canonical link) clustering hospitals and periods.

^c^ Percentages were calculated from the total number of people in the group.

### Secondary Outcomes

The incidences of secondary end points were lower in group 24 than in group 48 besides those of other infectious diseases. In the ITT population, 14 SSIs occurred in group 24 (2.2%) and 19 in group 48 (3.3%). The difference in SSI occurrence was −1.08 percentage points (95% CI, −2.98 to 0.83 percentage points), showing noninferiority of group 24 when the 4-percentage-point margin was used (Table 2). The results of the adjusted ITT analyses, per-protocol analysis, and analysis in per designated procedure population were all consistent with those of the ITT analyses when the 4-percentage-point margin was used (Figure 2B). The crude estimates of SSI within 30 days after surgery were not significantly different between group 24 and group 48 (HR, 0.67; cluster-robust 95% CI, 0.30-1.50) (Figure 3B). Subgroup analyses did not show any advantage of the longer AMP in group 48 (eFigure 4 in Supplement 2).

In the ITT population, 11 UTIs (1.7%) were observed in group 24 vs 13 UTIs (2.2%) were observed in group 48 (risk difference of UTI, −0.51 percentage points [95% CI, −2.53 to 1.51 percentage points]); 3 RTIs (0.5%) in group 24 vs 5 RTIs (0.9%) in group 48 (risk difference of RTI, −0.39 percentage points [95% CI, −1.41 to 0.63 percentage points]); and 3 other infectious diseases (0.5%) in group 24 vs 2 other infectious diseases (0.3%) in group 48 (risk difference of other infectious disease, 0.13 percentage points (95% CI, −0.34 to 0.60 percentage points) (Table 2). Mortality was observed in 2 participants in group 48, both of whom died because of aspiration pneumonia with severe emphysema. No life-threatening cardiovascular event was observed in either group within 30 days after surgery.

The rates of remaining hospitalized after surgery were comparable between group 24 and group 48 (Table 2). There was no significant between-group difference in the crude estimates of hospitalization within 30 days after surgery (HR, 1.17; 95% CI, 0.86-1.60) (eFigure 5 in Supplement 2). There were 3 participants (0.5%) in group 24 and 4 (0.7%) in group 48 who were readmitted in the hospital within 30 days after surgery for non-HAI reasons (eTable 4 in Supplement 2). Two patients, 1 from each group, experienced UTI after readmission.

Two participants with SSI were positive for methicillin-resistant *Staphylococcus aureus* (MRSA) and 4 participants with UTI were positive for extended-spectrum beta-lactamase-producing *Escherichia coli* (eTable 2 in Supplement 2). The difference in the risk of antibiotic resistance to HAI pathogens (group 24 vs group 48) in the ITT population was −0.38 percentage points (95% CI, −1.31 to 0.59 percentage points) (Table 2).

## Discussion

In this trial, AMP discontinuation within 24 hours was noninferior to longer duration in preventing HAI within 30 days after clean orthopedic surgery. Moreover, this was achieved without any signs of prolonged hospitalization, increased antibiotic resistance, or serious adverse events. To our knowledge, this is the first large size clinical trial to elucidate the effect of AMP duration on postoperative HAI in the field of orthopedic surgery. Our results support the global notion against antimicrobial resistance and may encourage surgeons to shorten AMP duration and reduce the antibiotic load in clean orthopedic surgeries.^11^

Unnecessary prolongation of the AMP should be discouraged because of the potential risk of adverse events^11,12,17^ and antibiotic resistance.^4^ Although we found no increase in resistance in group 48 compared with group 24, it is generally considered that even small increases in antibiotic use could be problematic, and the additional cost and other side effects for longer AMP should not be ignored.^11^ The high resistance of *S. aureus* is an important issue observed worldwide,^18^ with the United States and Japan being countries with the highest rates of resistance. Therefore, the Japanese Ministry of Health, Labor and Welfare launched a national action plan to reduce the rate of MRSA from 51% to less than 20% by 2020 (results pending as of this writing), while reducing the total amount of antibiotics used.^19^ Our findings support this aim, and may encourage nearly half^13^ of the orthopedic surgeons to adopt a shortened AMP duration.

The new CDC^3^ and WHO^5^ guidelines recommended no additional dose after wound closure. Shorter AMP duration may promote patients’ early rehabilitation and also reduce the amount of work of the health care team.^7^ However, several concerns exist in the field of orthopedic surgery regarding this recommendation. First, several societies have challenged this recommendation, because of the limited evidence.^7,8,20^ Second, several studies have indicated the possibility of a higher SSI risk when AMP duration is too short.^17,21,22^ As per the WHO guidelines, when the quoted studies were limited to clean orthopedic and cardiovascular surgeries,^21,23,24,25,26,27^ the pooled risk ratio of an additional dose after surgery significantly favored postoperative AMP.^16^ A similar trend was observed for orthopedic surgeries quoted in the CDC guidelines,^23,24,28,29,30,31^ although not significant.^16^ In contrast, several domestic guidelines^6,10^ permitted an even longer duration, up to 48 hours after surgery, for several orthopedic implant-related procedures, as there was insufficient evidence to recommend discontinuation within 24 hours after surgery. A recent large-scale retrospective study showed that better outcomes may be expected by selecting high-risk patients with long-term use of antibiotics following arthroplasty.^32^ Because of these concerns, most of our participating physicians still considered it ethically inappropriate to conduct a comparison study to compare the effect of intraoperative AMP against any other in our field. Therefore, we have conducted a comparison study indicating the inefficacy of longer durations. Thus, further studies are necessary to verify the effect of intraoperative AMP in the orthopedic fields.

The relatively few crossovers without any loss to follow up for HAI within 30 days of surgery in the analyzed cohort, may have strengthened our results. More than 94% of the study population was evaluated in person by the physicians, without a postal or telephone survey. Furthermore, more than 60% was still hospitalized on postoperative day 14, which is the relatively reported median onset time of SSI, RTI, and UTI (13-15 days,^33,34^ 4-6 days,^35,36,37^ and 8-11 days,^35,36,37^ respectively), after orthopedic surgery. Among Organization for Economic Co-operation and Development countries, Japan has the longest hospital stays in acute care.^38^ This feature of the Japanese health care system enabled us to examine a large proportion of our participant population during their hospitalization and made missing of any out-of-hospital outcomes substantially less likely in this study.

### Limitations

This study had some limitations. First, the participants’ allocation could not be perfectly masked from the participants because of the nature of the study design. Although participants did not know the group to which they were allocated at the time of undergoing the surgery, they could recognize it 24 hours after the surgery when they received additional antibiotics. Second, the allocation schedule was announced to the participating hospitals before the study to provide a reasonable time to minimize human errors and crossovers regarding new AMP protocols. Although we encouraged them not to select patients according to our allocation schedules to minimize surgeons’ preferences, selection bias could not be excluded. The characteristics of the study groups were mostly similar, with the number of surgeries performed in both groups constantly increasing without a statistical difference throughout the study period, thus ensuring comparability of the 2 groups. Moreover, no data indicated any advantage for group 48 according to our subgroup analyses of various variables. Therefore, the presence of unknown bias that could challenge the noninferiority of the shorter AMP may be less likely. Third, the interval and number of additional doses after the initial AMP may have varied among procedures. Although the AMP duration was strictly controlled with relatively few crossovers, the variation in the total antimicrobial dose administered before discontinuation in the 2 groups was not considered in our results. Fourth, patient care may be different in Japan compared with other countries. In this trial, more than 60% of the patients had a urinary catheter in place for more than 24 hours, and the drains were left in site for more than 48 hours in 40% of the patients. Although balanced between the 2 groups, these practices may be different in some countries, which bears consideration in applying our results. Fifth, although there was no significant difference among participating institutions in our subgroup analyses, approximately 50% of patients were from a single center. Sixth, the short follow-up period for implant-related surgery may underestimate the true incidence of SSI. Currently, the CDC recommended a minimum of 90-day follow-up for implant-related procedures.^39^ The lack of late SSIs that have occurred after 30 postoperative days may have attenuated our results. Seventh, the primary outcome of this study was the cumulative incidence of all postoperative bacterial infectious diseases which required antibiotic therapy within the 30-day period after surgery. Therefore, the surveillance definitions of UTI and RTI used in this study may differ in other circumstances.

## Conclusions

We found that AMP discontinuation within 24 hours after wound closure was noninferior to longer duration in preventing postoperative HAI. This was achieved without any signs of prolonged hospitalization or increased antibiotic resistance within 30 days after clean orthopedic surgery. Our findings support the global aim against antimicrobial resistance, which may reduce the socioeconomic burden, especially in institutions where prolonged AMP duration is still implemented. Further studies are necessary to determine whether AMP duration could be further shortened in our field, as recommended by the CDC and WHO guidelines.

## Notes

Supplement 1.
Trial Protocol

Supplement 2. **eTable 1.** Unified Surgical Site Infection Prevention Protocols **eTable 2.** Detailed Information of Patients With Healthcare-Associated Infections **eTable 3.** Primary and Secondary Endpoints of the Culture Positive Patients in the Intention-to-Treat Population **eTable 4.** Detailed Information of Patients Who Readmitted the Hospital Within 30 Days After Surgery **eFigure 1.** Time Course of Participants’ Recruitment **eFigure 2.** Time Course of Follow-up Rate During the 30−180 Days After Surgery **eFigure 3.** Forest Plot of Risk Ratios (95% Confidence Interval [CI]) for Healthcare-Associated Infections by Subgroups (Group 24 Versus Group 48) **eFigure 4.** Forest Plot of Risk Ratios (95% Confidence Interval [CI]) for Surgical Site Infections by Subgroups (Group 24 Versus Group 48) **eFigure 5.** Probability of Hospital Stays in Group 24 and Group 48 During 30 Days After Surgery

Supplement 3.
Nonauthor Collaborators

Supplement 4.
Data Sharing Statement
