# Supplementary material for: Effect of Antimicrobial Prophylaxis Duration on Health Care–Associated Infections After Clean Orthopedic Surgery: A Cluster Randomized Trial
Source: JAMA Netw Open. 2022 Apr 12;5(4):e226095. doi: 10.1001/jamanetworkopen.2022.6095 (PMC9006110; doi:10.1001/jamanetworkopen.2022.6095)
Supplement: Supplement 1. — Trial Protocol [file jamanetwopen-e226095-s001.pdf]

Date 2017/11/1

Clinical Trial Protocol

Prevention of postoperative orthopedic infection. Multicenter prospective comparative study

Principal Investigator

Hiroshi Okazaki, General Manager, Vice President

Kanto Rosai Hospital, Department of Orthopedics and Spine Surgery

〒 211-8510

Address: 1-1-1 Sumiyoshi-cho, Nakahara-Kizuki, Kawasaki City, Kanagawa Prefecture

TEL 044-411-3131 FAX 044-433-3150

E-mail okazarov@b-star.jp

Research Secretariat

Koji Yamada

Chief, Department of Orthopedics and Spine Surgery, Kanto Rosai Hospital

〒 211-8510

Address: 1-1-1 Sumiyoshi-cho, Nakahara-Kizuki, Kawasaki City, Kanagawa Prefecture

TEL 044-411-3131 FAX 044-433-3150

E-mail forpatients2008@gmail.com

Planned period of clinical research: From the date of approval by the Ethics Committee to 180 days after the completion of patient registration

First draft: February 1, 2017 Version 1.0

Revision: November 1, 2017 Version 1.2

Table of Contents

Table of Contents p 2

Summary p 3–7

1 Purpose and importance p 8

2 Background and the scientific rationale for the study p 8–12

3. Selection Policy for Research Subjects p 12–13

4 Method and duration of the research p 13–16

5 Survey Items and Methods p 16–19

6. Statistical analysis p 19–21

7 Registration and Data Collection p 21–23

8 Informed Consent p 23–25

9. Publication of research results p 25

10. Personal Information, etc. p 25

11 Proper handling of personal information p 26–27

12 Burden, anticipated risks (including possible adverse events), and benefits to the research subjects. Comprehensive evaluation of these and measures to minimize burden and risk p 27

13 Conflicts of interest related to the funding source of the research, the research of the research institution, and the earnings of the individual, Status of Conflicts of Interest Related to Research by Researchers, etc. p 27

14. Intellectual Property p 28

15 Public announcement p 28

16 Method of reporting to the head of the research organization p 28

17 Response to consultation, etc., from research subjects, etc., and their related persons p 29

18 Financial burdens or rewards for the research subjects p 29

19 Handling of research results (including incidental findings) pertaining to research subjects when there is a possibility of important insights into the health of the research subjects, genetic characteristics that can be passed on to their offspring, p 29

20 Description of business, method of supervision of contractors p 29

21 The possibility that the information will be used for future research that is not identified at the time the consent is received. / Possibility of the information to be provided to other research institutions p 29

22 Provision of samples and information, to the collaborating institutions of this research or to other research institutions. p 30

23 Provision of samples, information, etc. to commercial organizations, etc. p 30

24 Response to adverse events p 30

25 Handling of deviations from the initial plan p 31

26 Changes to the research protocol p 31

27 Research organization p 32

Summary

Summary of the research protocol

<u>Title of the clinical research</u>

Non-inferior comparative study comparing one or two day antimicrobial prophylaxis after clean orthopaedic surgery (NOCOTA study): a study protocol for a cluster pseudo-randomized controlled trial comparing duration of antibiotic prophylaxis

<u>Purpose of the study</u>

To compare the efficacy of antimicrobial prophylaxis (AMP) administered within 24 hours postoperatively with those administered between 24 and 48 hours in patients undergoing clean orthopedic surgery. The primary outcome will be the cumulative incidence of all postoperative bacterial infectious diseases which will be diagnosed within 30 days (the operation date being defined as "Day 0") after surgery and requiring antibiotic therapy. Secondary endpoints will include the incidence of surgical site infection (SSI), urinary tract infection, respiratory tract infection, and other infections, death within 30 days after surgery, cardiovascular events, prolonged hospitalization for more than 30 days after surgery, and the emergence of antimicrobial resistance of bacteria causing postoperative infections. Therapeutic antimicrobial administration refers to cases in which antimicrobial agents were administered for the purpose of treating postoperative complications when bacteria are presumed to be the cause of infections are included. Therefore, cases in which antimicrobial agents were mistakenly administered for influenza or norovirus infections will not be included. The date of the infection onset shall be the date when the administration of therapeutic antimicrobial agents is necessary, not the date when the administration of therapeutic antimicrobial agents was initiated.

<u>Research Design</u>

Multicenter, prospective, cluster-randomized, controlled, non-inferiority study

Target number of cases

500 for each group, total of 1000 cases

The table below shows the target number of cases for each group.

Group	Target number of cases
Group 24; AMP administered within 24 hours	500
Group 48; AMP administered over 24 hours and within 48 hours	500

<u>Target</u>

Hospitalized undergoing clean orthopedic surgery

<u>Inclusion criteria</u>

Patients undergoing inpatient surgery performed by a physician in charge of orthopedic surgery in the Department of Orthopedic Surgery, Department of Rheumatology, and Department of Emergency Medicine at a facility registered to participate in this study who meet the conditions 1) through 6) below.

1) Aged 20 years or older at the time of obtaining consent.
2) Hospitalized patients undergoing orthopedic surgery by orthopedic surgeons who belong to orthopedics, rheumatology, or emergency department.
3) Clean surgery*.
4) Ability to read and understand Japanese.
5) Oral or written consent for participation obtained from the individual or a substitute (relative).
6) Had a primary intention wound closure.

*Surgery defined as Class I (clean surgery) in the CDC wound classification.

Exclusion criteria

If any of the following exclusion criteria are met, the subject will not be included in the study.

1) When part of the fixed implant is exposed to the outside of the body (e.g., external fixation, percutaneous pinning, etc.)
2) Amputation surgery
3) Needle biopsy
4) Implant removal
5) Reconstructive surgery involving skin tissue such as flap surgery
6) Antimicrobial, antiviral, antifungal and anti-tuberculosis drug therapy is given at the time of surgical decision.
7) Percutaneous vertebroplasty (Balloon Kyphoplasty, Vertebral Plasty, etc.)
8) Surgery performed in collaboration with other departments

Discontinuation criteria

1) When the antibacterial, antiviral, antifungal and antituberculosis drug are used as treatment on the day of surgery.
2) When the physician in charge determines that it is not appropriate for the subject to continue the study due to the occurrence of adverse events, worsening of complications, or worsening of the primary disease.
3) Found to be ineligible as a subject after the start of this study, or discharged within 48 hours of surgery
4) When the subject wishes to discontinue the study.
5) When the physician in charge judges that the continuation of this research is impossible or unsuitable for reasons other than the above.

Antimicrobial agents used

(1) Cefazolin (1st generation cephalosporin antibacterial agent)

(2) Vancomycin (Glycopeptide antimicrobial, used for cephalosporin allergic patients)

(3) Clindamycin (Lincomycin antibiotic, used for patients with cephalosporin allergy)

Dose and method of administration

[Group 24; AMP administered within 24 hours]: Cefazolin 2g will be administered preoperatively for the first time. Additional intraoperative doses of 1 g of cefazolin will be administered according to the additional dosing intervals recommended in the "Practical Guidelines for the Appropriate Use of Antimicrobial Agents in the Prevention of Postoperative Infections" established by the Japanese Society for Chemotherapy and the Japanese Society for Surgical Infectious Diseases, according to renal function. The additional postoperative dose should be completed within 24 hours after surgery.

[Group 48; AMP administered over 24 hours within 48 hours]: Cefazolin 2g will be administered preoperatively for the first time. Additional intraoperative doses of 1 g of cefazolin will be administered according to the additional dosing interval recommended in the "Practical Guidelines for the Appropriate Use of Antimicrobial Agents for the Prevention of Postoperative Infections" established by the Japanese Society for Chemotherapy and the Japanese Society for Surgical Infectious Diseases, according to renal function. Additional postoperative doses should be given at least 24 hours and no more than 48 hours after surgery.

Observation period

30 days after surgery

Evaluation Items

Effectiveness

① Primary endpoints

The total incidence of complications (SSI, urinary tract infection, respiratory tract infection, and other infections) requiring postoperative antimicrobial therapy (excluding antivirals, antifungals, and antitubercular agents)" within 30 days after surgery (the day of surgery is defined as day 0).

② Secondary endpoints

Percentage of SSIs, percentage of urinary tract infections, percentage of respiratory tract infections, percentage of other infections, percentage of deaths, percentage of cardiovascular events, percentage of continuous hospitalization for 30 days after surgery, and percentage of resistance of bacteria causing postoperative SSIs within 30 days after surgery

Safety

Adverse events: subjective symptoms, other findings, anaphylaxis

Observation and inspection

Observations, test items, and clinical research schedule:

Refer to "Table 1: Observation, Inspection, and Evaluation Items and Timing".

Contraindicated drugs

None in particular

Research period

From Ethics Committee approval date to 180 days after completion of patient registration

Research Organization

Principal investigator Research secretariat

Kanto Rosai Hospital, Department of Orthopedics and Spine Surgery

Orthopaedic Surgical Site Infection (OSSI) Study Group Secretariat:

Tel 044-411-3131 (Hospital Representative)

Koji Yamada, Chief of Orthopaedic Surgery

Hiroshi Okazaki, Vice President, General Manager of Orthopaedic Surgery

Department of Orthopaedic Surgery, School of Medicine, The University of Tokyo

Kosei Nagata, Orthopaedic Surgery

Department of Orthopedics, Tokyo Metropolitan Hiroo Hospital

Tel: 03-3444-1181 (hospital representative)

Yasuhito Tajiri, Chief of Orthopaedic Surgery

Head of Statistical Analysis

Department of Medical Research and Management for Musculoskeletal Pain, Graduate School of Medicine, The University of Tokyo

Takayuki Oka, Specially Appointed Associate Professor

Department of Biostatistics, Division of Health Sciences and Nursing, Graduate School of Medicine, The University of Tokyo

Tomohiro Shinozaki, Assistant Professor

7-3-1, Hongo, Bunkyo-ku, Tokyo 113-0033 , Japan TEL : 03-3815-5411

Research Schedule

Table 1 Observation and evaluation items and timing

	Research period

Item		Preliminary inspection Period		Medication period + Post observation period						
		Pre-registration	Registration	operation	+1 day	+2 days.				30 days, or At the time of discontinuation
Obtaining Consent		○								
Patient background		○								
Eligibility check		[d]○	○							
Blood examination		[e]△		[f]△	[f]△					[f]△
X-rays		[e]△		[f]△						[f]△
Registration and allocation			←→	←→						
Antimicrobial administration	cefazolin			○[g] ←→	←→	←→				
	vancomycin			○[g] ←→	←→	←→				
	clindamycin			○[g] ←→	←→	←→				
Subjective &other findings			[e]○	○						○
Adverse events				←→	←→	←→	←→	←→	←→	←→
[a]SSI										○
[a]Urinary tract infection										○
[a]Respiratory infection										○
[b]Other Infections										○
[c]Death and cardiovascular event										○
[c]Hospitalization										○
[c]Rehospitalization after discharge										○

[a] Based on the modified definition of the U.S. Centers for Disease Control and Prevention guideline.

[b] Based on clinical diagnosis

[c] Investigate only events within 30 days of surgery.

[d] Observations are made to confirm eligibility.

[e] Perform if no appropriate tests have been performed immediately before.

[f] If necessary, the same tests as in the regular postoperative examination will be performed. Thereafter, samples should be taken when considered clinically necessary.

[g] Use cefazolin if not allergic to beta-lactams, vancomycin or clindamycin if allergic to beta-lactams .

Study period: From the time consent is obtained until 30 days after surgery (or at the time of discontinuation)

Enrollment and allocation: Conducted during the observation period.

1 Purpose and Importance

The purpose of this study is to compare the efficacy of antimicrobial prophylaxis (AMP) administered within 24 hours postoperatively with those administered between 24 and 48 hours in hospitalized patients undergoing clean orthopedic surgery. The primary endpoint will be the total incidence of health care–associated infections (HAIs) including surgical site infection (SSI), urinary tract infection, respiratory tract infection, and other infections, requiring postoperative antimicrobial therapy (excluding antivirals, antifungals, and antitubercular agents) within 30 days after surgery (the day of surgery will be defined as day 0). The secondary endpoints will be the incidence of SSI, urinary tract infection, respiratory tract infection, and other infections, death, and cardiovascular events within 30 days after surgery, the incidence of continuous hospitalization for 30 days after surgery, and the incidence of resistant strains of bacteria causing postoperative SSI. These studies will clarify the usefulness of the duration of AMP administration. Therapeutic antimicrobial administration refers to the administration of antimicrobial agents for the purpose of treating postoperative complications, and only when bacterial infection is suspected. Therefore, cases in which antimicrobial agents were mistakenly administered for influenza or norovirus infections are not included. The date of onset of the infection shall be the date of onset of the infection that necessitated the administration of therapeutic antimicrobial agents, not the date when the administration of therapeutic antimicrobial agents was initiated.

To our knowledge, there is no study using composite infectious endpoints comparing the efficacy on duration of AMP. This study will provide clinically meaningful information on the impact of duration of AMP on postoperative HAIs and other events. In order to provide safer surgeries for patients, this study will provide important information on the appropriate duration of antimicrobial administration, and is expected to provide high-quality information that has been lacking in the development of guidelines.

2 Background and the scientific rationale for the study

2.1 Background

Problems with conventional preventive studies regarding AMP duration

Perioperative AMP use has the highest level of evidence for the prevention of SSI and is strongly recommended in various guidelines. In the field of orthopedic surgery, there are several high-quality studies on various surgical procedures, and all of them have consistently shown its usefulness. However, although the evidence for the administration of prophylactic antimicrobial agents is high, the evidence for the duration of prophylactic antimicrobial agents is still weak, especially in the field of orthopedics.

The recommended duration of AMP administration is becoming shorter worldwide, mainly in terms of preventing resistant organisms, and is generally recommended to be administered within 24 hours. The U.S. Centers for Disease Control and Prevention's (CDC) new guidelines for SSI prevention recommend only intraoperative AMP administration for all procedures, which is even shorter. However, the "Practical Guidelines for the Appropriate Use of Antimicrobial Agents for the Prevention of Postoperative Infections," compiled by the

Japanese Society for Chemotherapy in 2016, subdivided and examined in detail each surgical procedure, and found that additional AMP administration should rather be recommended for more than 24 hours after some orthopedic procedures. Furthermore, in the field of orthopedic surgery, there is no strong evidence that not giving additional postoperative AMP doses reduces the incidence of SSIs. There are no high-quality studies on the usefulness and safety of dosing within 24 hours compared with dosing over 24 hours. And the evidence for the usefulness of dosing within 24 hours is still weak. In addition, the ″Practical Guidelines for the Appropriate Use of Antimicrobial Agents for the Prevention of Postoperative Infections″ recommends administration within 48 hours in some instrumentation procedures, but this is not because there is evidence that administration within 48 hours improves the rate of SSI compared with administration over 48 hours, but because the rate of resistant bacteria in all infections, including those other than SSI, may increase with administration over 48 hours. This is not because there is evidence that administration within 48 hours improves the SSI rate more than administration for more than 48 hours, but rather because of foreign data showing that the incidence of resistant strains in all infectious diseases, including non-SSI, may increase with administration for more than 48 hours. But, there is no high quality evidence if this is also the case in Japan. Although the usefulness of prophylactic administration of antimicrobial agents has been shown at a high level of evidence in the field of orthopedic surgery, the appropriate period of prophylactic administration has not been sufficiently verified, which is an essential issue for providing safer surgery to patients in the future.

In this study, we will conduct a multicenter prospective comparative study to establish high-quality evidence on the appropriate duration of administration in clean orthopedic surgery, using the multicenter research groups that have been working to prevent SSIs.

Approaches to SSI research at the Society for Orthopaedic Surgical Site Infection (OSSI)

A multicenter prospective SSI database was started in November 2013 by a research group; Society for Orthopaedic Surgical Site Infection [OSSI) Research Group. consisting of seven facilities of the Rosai and Tokyo Metropolitan Hospitals, which serve as regional core functions. About 350 surgeries are registered every month, and the number of cases now exceeds 10000. We have published more than 20 conference reports and many original papers in Japan and abroad, and have received several awards.

Previous data analysis has shown that approximately 4.2% infectious events occur postoperatively in clean orthopedic surgery, including not only SSIs but also various other infections (Koji Nakajima et al., 2016 Japanese Society of Bone and Joint Infection Award).SSIs are difficult to diagnose, and is difficult to rule out when febrile events occur. Therefore, in practice, postoperative SSIs are kept in mind in about 4.2% of cases, and patients are monitored with concern.

All of these infectious events are complications that may result as life-threatening events. Particularly common are postoperative urinary tract infections. All postoperative urinary tract infections occurred in patients with indwelling urinary catheters, and furthermore, the incidence tended to increase (P<0.01) as the duration of indwelling catheters increased (Kazuhiro Kiba et al., 2016 Japanese Society of Bone and Joint Infections). Urinary tract infections are the most common healthcare-associated infection, accounting for more than 30% of infections in acute care facilities (Klevens 2007), and are known to increase readmission rates and 30-day

mortality rates (Wald 2008). The same is true for postoperative respiratory infections, which are considered a risk for early postoperative mortality. Thus, these infections are sometimes a matter of life-threatening events for patients, and perioperative measures should be aimed at preventing not only SSIs but all these infections together. Prophylactic antimicrobial therapy has been shown to be useful not only for SSIs but also for prevention of urinary tract and respiratory tract infections, and the duration of therapy may have an impact on these infections in general. However, there are no studies that have examined the pros and cons of the duration on antimicrobial prophylactic by considering the occurrence of these multiple infections as a composite endpoint.

The recommended duration of AMP administration is in the trend of recommending within 24 hours worldwide. On the other hand, the Japanese "Guidelines for the Prevention of Postoperative Infections in Bone and Joint" issued in 2015 recommended administration discontinuation within 48 hours. There is no strong evidence to support AMP administration for more than 24 hours in the field of orthopedics. The guidelines are based on the current situation in Japan, where many orthopedic surgeons still prefer administration for more than 24 hours. Shortening the duration of AMP administration not only leads to early leaving the bed, but also reduces medical costs, workload of co-medical staff, nor emergence of resistant bacteria. Therefore, even if the efficacy in preventing SSIs is the same for administration within 24 hours as for administration over 24 hours, if the safety is the same, it can be recommended to shorten the administration period with more confidence in Japan. When considering postoperative infections as a whole, there is still a concern that short-term administration (administration within 24 hours) may lead to a higher risk of various infections. However, to our knowledge, no studies have explored this issue, and the pros and cons remain unclear.

In 2016, "the Action Plan on Drug Resistance" has been launched in Japan (http://www.mhlw.go.jp/stf/seisakunitsuite/bunya/0000120172.html). According to this plan, the total amount of antimicrobial agents used in Japan is to be reduced by 33% by 2020. In the field of orthopedic surgery, the most common use of antimicrobial agents is the administration of prophylactic antimicrobial agents during surgery. Shortening the administration period of prophylactic antimicrobial agents is expected to lead to a significant reduction in the amount of antimicrobial agents used in the field of orthopedics, and may make a significant contribution to this action plan. In addition, shortening the duration is generally considered to improve the incidence of resistant bacteria. The action plan includes an effort to reduce the proportion of methicillin-resistant Staphylococcus aureus (MRSA) in Japan (51% in 2014 as indicated by the World Health Organization (WHO)), to 20% or less by 2020. SSIs are the predominant infection in the field of orthopedics, and most of the causative organisms are *Staphylococcus aureus*, and moreover, most of them are usually resistant organisms, MRSA. Shortening the duration of AMP may not only significantly reduce the amount of antimicrobial use, but also improve the proportion of MRSA in the overall SSI. Therefore, the results of this study may present a very important outcome for the government to achieve the major goals mentioned above, and may become one of the actual measures that the government should promote in the future.

2.2 Scientific rationale for the study

Cluster randomized prospective comparative study on duration of antimicrobial prophylaxis

Based on the above background, we will investigate the effect on duration of AMP on the prevention of

postoperative HAIs in clean orthopedic surgery. Specifically, the study will be conducted as follows.

1) Comparison of the incidence of postoperative infectious events (composite events) between the group treated within 24 hours after surgery (Group 24; short-term group) and the group treated between 24 and 48 hours after surgery (Group 48; long-term group)

The antimicrobial agents to be used are mainly cefazolin (a first-generation cephalosporin), which is the first-line drug of choice in almost all SSI prevention guidelines. Only when penicillin- or cephalosporin-allergic patients cannot use cefazolin, vancomycin or clindamycin, which are recommended as alternatives, will be used. Previous studies have shown that the antimicrobial agent used in OSSI investigators is cefazolin in 98% of cases, indicating that the global standard antimicrobial agent have been selected in most cases. Therefore, we believe that there is no particular need to change our usual practice in selecting the antimicrobial agents. In this study, we will divide the patients into two groups and compare only the duration of administration. In cluster randomization, the participating institutions will be divided into two groups, Group A and Group B. Group A will be administered within 24 hours first for a certain period of time, and Group B will be administered between 24 and 48 hours at the same time. After a certain observation period, Group A will be switched to 24 hours or more but less than 48 hours, and Group B will be administered 24 hours or less. The switch of the dosing period will be considered as one cycle, and this process will be repeated for several cycles, until the planned number of recruited patients is collected. After all recruitment, we will compare the primary endpoints of the data obtained. By calculating the sample size based on the previous activities of OSSI study group, we are confident that this study can be sufficiently validated by recruiting patients for 6 months to 1 year, and that the patient background will be almost the same with the planned allocation method.

2) Comparison of the incidence of various postoperative events between Group 24 (short-term group) and Group 48 (long-term group)

As mentioned above, only “the duration” will be compared between the two groups. In the cluster randomization method, the participating centers will be randomly divided into two groups (Group A and Group B) at a certain time of the year (e.g., January to March), and Group A will be administered within 24 hours for a certain period of time, while Group B will be administered between 24 and 48 hours at the same time. When the time for switching arrives (March in the above example), the institutions will be randomized again and the institutions will be reassigned to Group A (within 24 hours) and Group B (between 24 and 48 hours). Such re-randomization will be repeated until the planned number of recruited patients is collected. When we reach the planned number of recruited patients, the secondary endpoints of the data obtained will be compared. The timing of the re-randomization will be determined based on feasibility before the start of the study.

By conducting this study, the relationship between the duration of antimicrobial administration and the effectiveness of prevention of postoperative infectious events will be clarified with sufficient statistical power. In addition, the study evaluating each of the various events is likely to be underpowered considering the original rate of occurrence of each individual event, but the results obtained may differ from the commonly accepted notion. It is possible to profile the duration of prophylactic antimicrobial therapy and its effect on the prevention

of various events, which will provide very useful information not only to orthopedic surgeons but also to government policy makers on measures to prevent SSIs and other perioperative complications, which are currently an urgent issue.

The ethics committee will review the appropriateness of conducting this study from the perspective of ethical, scientific, and medical validity, and the study will be conducted with the approval of the ethics committee of all participating hospitals.

3 Selection Policy for Research Subjects

3.1 Eligibility criteria

Inpatients undergoing surgery performed by a physician in charge of orthopedic surgery in the Department of Orthopedic Surgery, Department of Rheumatology, and Department of Emergency Medicine at a facility registered to participate in this study who meet the conditions 1) through 6) below.

1) Aged 20 years or older at the time of obtaining consent.
2) Hospitalized patients undergoing surgery performed by orthopedic surgeons of orthopedics, rheumatology, or emergency department.
3) Clean surgery*.
4) Ability to read and understand Japanese.
5) Oral or written consent for participation obtained from the individual or a substitute (relative).
6) Had a primary intention wound closure .

*Surgery defined as Class I (clean surgery) in the CDC wound classification.

[Rationale]

1,2,4,5) To prevent patients who lack capacity to consent from participating in research

3) To include only clean surgery

6) For efficacy evaluation and safety considerations

3.2 Exclusion criteria

If any of the following exclusion criteria are met, the subject will not be included in the study.

1) When part of the fixed implant is exposed to the outside of the body (e.g., external fixation, percutaneous pinning, etc.)
2) Amputation surgery
3) Needle biopsy
4) Implant removal
5) Reconstructive surgery involving skin tissue such as flap surgery

6) Antimicrobial, antiviral, antifungal and anti-tuberculosis drug therapy is given at the time of surgical decision.
7) Percutaneous vertebroplasty (Balloon Kyphoplasty, Vertebral Plasty, etc.)
8) Surgery performed in collaboration with other departments

[Rationale]
1,2,5,6,8) For efficacy evaluation and safety considerations
3,4,7) Because the incidence of surgical site infections is extremely low and the procedure is not a major problem

3.3 Discontinuation criteria

4) When the antibacterial, antiviral, antifungal and antituberculosis drug are used as treatment on the day of surgery.
5) When the physician in charge determines that it is not appropriate for the subject to continue the study due to the occurrence of adverse events, worsening of complications, or worsening of the primary disease.
6) Found to be ineligible as a subject after the start of this study, or discharged within 48 hours of surgery
4) When the subject wishes to discontinue the study.
5) When the physician in charge judges that the continuation of this research is impossible or unsuitable for reasons other than the above.

Antimicrobial agents used
(1) Cefazolin (1st-generation cephalosporin)
(2) Vancomycin (Glycopeptide, used for cephalosporin allergic patients)
(3) Clindamycin (Lincomycin, used for patients with cephalosporin allergy)

This research will be conducted in compliance with the " Ethical Guidelines for Medical and Health Research Involving Human Subjects" (http://www.mhlw.go.jp/file/06-Seisakujouhou-10600000-Daijinkanboukouseikagakuka/0000166072.pdf) (Ministry of Health, Labor and Welfare, Japan), and will be conducted in the spirit of the Declaration of Helsinki revised in 2008.

4 Method and duration of the research

4.1 Research Design

The study is a multicenter, cluster-randomized controlled trial with a non-inferiority test, consisting of a short-term group receiving prophylactic antimicrobials within 24 hours and a long-term group receiving prophylactic antimicrobials for more than 24 hours for patients undergoing inpatient surgery for clean orthopedic

surgery.

4.2 Outline of the research

The physician in charge obtains consent from patients who meet all the selection criteria and are not considered to be in conflict with any of the exclusion criteria. After obtaining informed consent, the physician confirms that the subject meets all the inclusion criteria and does not violate any of the exclusion criteria, enters the necessary information on the Web, and confirms the allocation results.

The physician in charge will start the study after confirming the allocation results on the web. Each subject will participate in the study for up to 30 days after consent is obtained. The details of each are shown in the figure below.

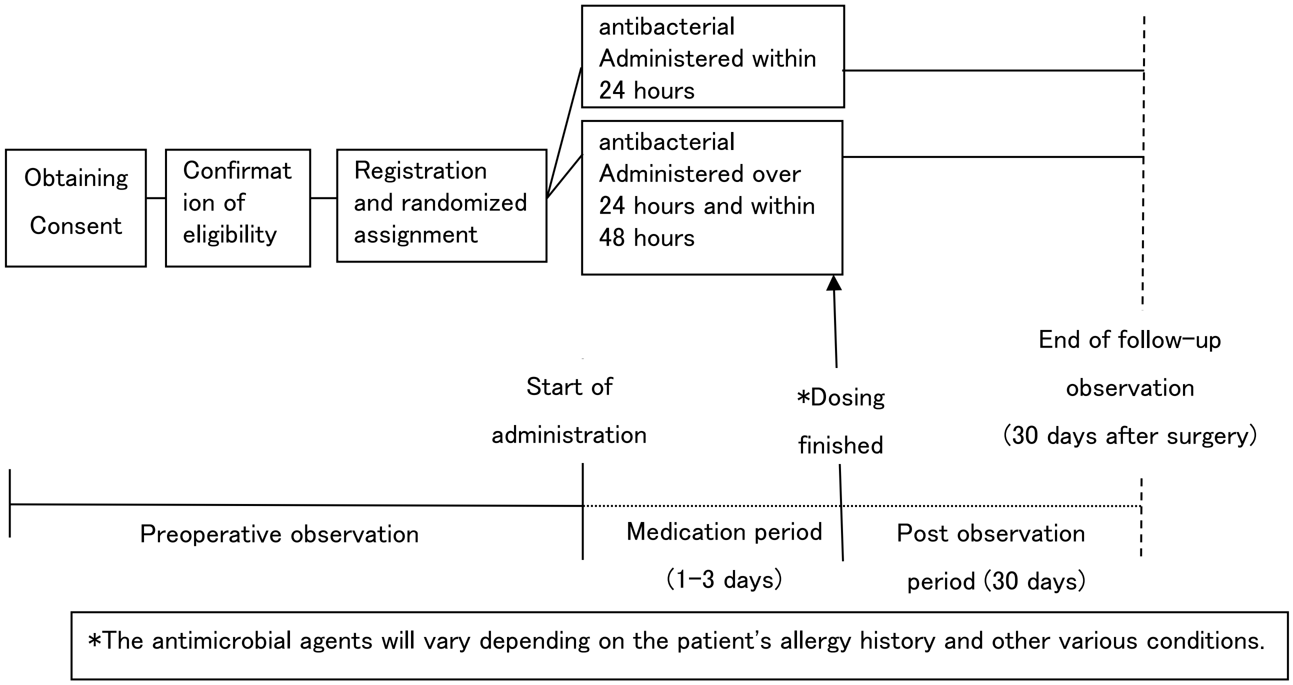

4.3 Expected duration of subject's participation in the study

30 days after surgery. If the patient dies within 30 days after the surgery, the period shall be up to the date of death.

4.4 Dosage and administration period of the drug used in this study

Dosage and administration of prophylactic antimicrobials should be in accordance with the documents described in the "DOSAGE AND ADMINISTRATION"of the package insert for each drug.

However, when cefazolin is used, the initial dose should be 2 g, and subsequent additional doses should be 1 g. After surgery, additional administration of prophylactic antimicrobial agents beyond the scheduled period for the purpose of SSI prophylaxis, including oral administration, should not be performed in principle. When infection is

suspected after surgery and antimicrobial agents are administered for therapeutic purposes, every effort should be made to identify the site and the causative organism of infection. In this case, there are no restrictions on the type and methods of antimicrobial agent to be used for therapeutic purposes.

4.5 Dosage form, content, properties, packaging, labeling, and storage method of the test drug

The test drug is listed below. Please refer to the attached document for detailed information.

1) Cefazolin

Product Name: Cefazolin Sodium for Injection 1g "Nichi-Iko".

Cefazolin Sodium for Injection 2g [Nichi-Iko] ®.

Manufactured and distributed by: Nichi-Iko Co.

Distributed by: Nichi-Iko Co.

Approval number: 1g 22500AMX00648000, 2g 22500AMX00649000

Dosage: 1g, 2g

2) Vancomycin

Product Name: Vancomycin Hydrochloride for Intravenous Infusion 0.5g [MEEK] ®.

Manufactured and distributed by: Kobayashi Kako Co.

Distributed by: Meiji Seika Pharma Co.

Approval number: 22700AMX00725000

Dosage: 0.5g

3) Clindamycin

Product Name: Clindamycin Phosphate Injection 600mg "NP

Manufactured and distributed by: Nipro Corporation

Approval number: 22700AMX00080

Dosage: 600mg

4.6 Provisions on concomitant medications (therapies)

(1) Contraindicated drugs

None in particular.

(2) Concomitantly restricted drugs

None in particular.

Subjects' compliance

(1) If an antimicrobial agent is prescribed by an other clinic where the surgery was not performed within 30 days

after the surgery, patients are asked to report it to the physician.

(2) During the study period, the patient is scheduled to come to the hospital according to the observation schedule.

(3) If the patient is unable to visit the outpatient clinic within 30 days after the surgery, the patient is asked to cooperate in confirming the condition by phone or mail method.

Survey of prior therapy, concomitant medications, and concomitant therapy status

The physician enrolling the patient in the study should be sure to check for any preoperative use of antibacterial, antiviral, antifungal, or antituberculosis medications.

4.7 Research period

From Ethics Committee approval date to 180 days after completion of patient registration

The registration will start after the approval of the ethics committees of all participating institutions and will last until the completion of 1000 surgical registrations.

The observation period should be from the time consent is obtained until 30 days after surgery (or at the time of discontinuation) ,however, if death occurs within 30 days after surgery, it should be until the date of death. The final evaluation will be performed at the postoperative examination after 30 days but within 180 days after surgery.

4.8 Participating Facilities

Name of medical institution	Address
Kanto Rosai Hospital	〒211-8510<br>1-1 Sumiyoshi-cho, Kizuki, Nakahara-ku, Kawasaki-shi, Kanagawa
Tokyo Metropolitan Hiroo Hospital	〒150-0013<br>2-34-10 Ebisu, Shibuya-ku, Tokyo
Tokyo Metropolitan Tama General Medical Center	2-8-29 Musashidai, Fuchu-shi, Tokyo 183-8524, Japan
Tokyo Metropolitan Institute of Gerontology and Gerontology	35-2, Sakae-cho, Itabashi-ku, Tokyo 173-0015, Japan
Cancer and Infectious Disease Center Tokyo Metropolitan Komagome Hospital	113-8677<br>3-18-22, Honkomagome, Bunkyo-ku, Tokyo

5. Survey items and methods

5.1. Survey Items

The physician in charge shall enter the subject identification code, date of obtaining consent, date of registration, and registration number in the case report form. In addition, the following items should be investigated and entered in the case report form from the date of obtaining consent to the date of registration.
1) Preoperative: age, month of birth, gender, DM status, dialysis status, rheumatoid arthritis status, disease classification, etc.
2) Postoperative: date of admission, date of surgery, height, weight, ASA classification, current smoking habit, urgent/standby, prophylactic antimicrobial (type), prophylactic antimicrobial (appropriate timing of initial administration), presence of multi-site surgery on the same day, time required for surgery, amount of blood loss, temperature at the end of surgery, number of surgeries, surgical procedure classification, etc.
3) After the next day: last dose of prophylactic antimicrobial (timing), urinary catheter placement, presence of postoperative drain placement, end of surgery – next morning maximum blood glucose level, etc.
4) SSI evaluation: date of last evaluation, presence or absence of SSI within the postoperative30 days, SSI diagnostic methods, etc.
5) SSI detail: Date of SSI occurrence, SSI depth, SSI evidence, date of culture specimen collection, SSI-causing organism, etc.
6) Complications: hematoma, reoperation (for treatment of SSI), ward debridement, cardiovascular and cerebrovascular disorders, respiratory tract infection and causative organism, urinary tract infection and causative organism, other infections (other than SSI, text entry) and causative organism, anaphylaxis, etc.
7) Post-discharge: outcome (date of discharge and date of death, if applicable), whether readmitted (date of readmission, if applicable), etc.

<u>Observations and test items for efficacy assessment</u>

Inspection items and inspection period

(1) Primary endpoints

Confirm and describe whether the patient had an SSI, urinary tract infection, respiratory tract infection, or other infection that required postoperative therapeutic antibacterial administration (excluding antivirals, antifungals, and antitubercular agents) within the postoperative 30-day period (the day of surgery is considered as day 0). They will be examined within the postoperative 180-day period.

(2) Secondary endpoints

Confirm and describe the occurrence of SSI, urinary tract infection, respiratory tract infection, other infections, death, cardiovascular events, continuous hospitalization for more than 30days, and emergence of resistant bacteria within the postoperative 30-day period. They will be examined within the postoperative 180-day period.

5.2. Method and observation period

Table 1 Observation and evaluation items and timing

Item		Research period								
		Preliminary inspection Period		Medication period + Post observation period						
		Pre-registration	Registration	operation	+1 day	+2 days.				30 days, or At the time of discontinuation
Obtaining Consent		○								
Patient background		○								
Eligibility check		[d]○	○							
Blood examination		[e]△		[f]△	[f]△					[f]△
X-rays		[e]△		[f]△						[f]△
Registration and allocation			⟷	⟷						
Antimicrobial administration	cefazolin			○[g] ⟷	⟷	⟷				
	vancomycin			○[g] ⟷	⟷	⟷				
	clindamycin			○[g] ⟷	⟷	⟷				
Subjective &other findings			[e]○	○						○
Adverse events				⟷	⟷	⟷	⟷	⟷	⟷	⟷
[a]SSI										○
[a]Urinary tract infection										○
[a]Respiratory infection										○
[b]Other Infections										○
[c]Death and cardiovasucular event										○
[c]Hospitalization										○
[c]Rehospitalization after discharge										○

[a] Based on the modified definition of the U.S. Centers for Disease Control and Prevention guideline.

[b] Based on clinical diagnosis

[c] Investigate only events within 30 days of surgery.

[d] Observations are made to confirm eligibility.

[e] Perform if no appropriate tests have been performed immediately before.

[f] If necessary, the same tests as in the regular postoperative examination will be performed. Thereafter, samples should be taken when considered clinically necessary.

[g] Use cefazolin if not allergic to beta-lactams, vancomycin or clindamycin if allergic to beta-lactams .

Observation period: from the time consent is obtained to the day after surgery30 (or at the time of discontinuation)

Allowable evaluation: The acceptable range of evaluation dates shall be as follows.

The evaluation date shall be within the following range, but shall not be canceled even if it is out of the acceptable range.

Assessment date of events within 30 days after surgery:

Events occurring within the postoperative 30-day period will be assessed within 180 days after the surgery.

6. Statistical Analysis

6.1. Sample size requirement

The required sample size is 500 cases per Group, total 1000 cases.

Rationale: Based on the analysis of the surgical outcomes of the previous survey of 2,589 cases, the rate of all postoperative infectious events was 4.2%. We calculated the sample size required to show non-inferiority by the Farrington-Manning test (a test of binomial proportions comparing 2 groups with a non-inferiority threshold as the null hypothesis) with the following setting.

- Power: 80%.
- Alpha (one-tailed test): 2.5%.
- Assumed risk of infection within 30 postoperative days in both groups: 1% to 8% ("P2" in the Figure) with alternative hypothesis of no-difference between Groups (specified as "D1 = 0" in the Figure).
- The non-inferiority threshold (null hypothesis, H_0): +1% to +5% ("D0" in the Figure).

The results are shown in the following Figure (horizontal axis [P2]: 30-day SSI risk, vertical axis [N1]: required sample size per group).

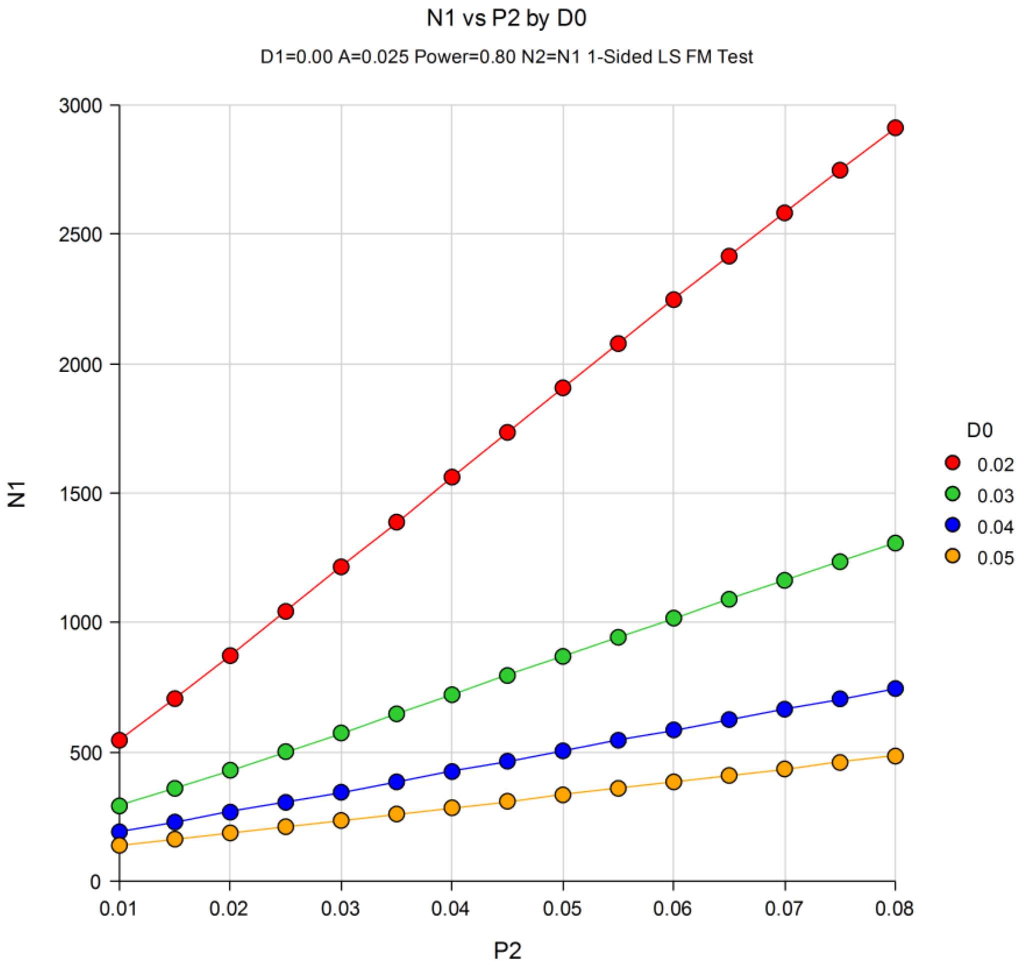

To show that the upper limit of the 95% confidence interval for the risk difference (risk in the short term group − risk in the long term group) does not exceed +4% (D0 = 0.04, blue line) for 30-day postoperative infections with 4% (P2 = 0.04) expected in the long term and short term groups, a study could be conducted with as few as 400 patients per group. To allow for the possibility of 10%–20% missing data due to subject dropout during the follow-up period, the required sample size was set at 500 cases per group.

6.2. Analysis population

For the analysis of the primary endpoint and all secondary endpoints, the analysis population will be all subjects who participate in the study.

6.3. Analysis method

1) Conduct both intention-to-treat (ITT) analysis and per protocol analysis.

Cases with missing values for the primary endpoint will be excluded from the analysis, and the ITT analysis will follow the comparison between the allocation group, despite of the adherence to allocated treatment (e.g., patients who are assigned to the short-term group but deviated from the allocation and received the long-term

treatment will still be on the short-term group, and *vice versa*). The analysis will exclude patients who deviated from the allocation and compare the short-term group with the long-term group in the remaining subjects.

2) Analysis method

(1) Primary endpoint: Infection within the first 30 postoperative days

Statistical method for hypothesis test: Farrington-Manning test.

The null hypothesis: the risk difference (risk in the short-term group − risk in the long-term group) is 4%.

Significance level (one-sided): 0.025

As an effect measure, we will calculate the risk difference with 95% confidence interval using generalized estimating equations (GEEs) for linear risk models that treat the time and facility corresponding to the assignment of treatments as "clusters".

(2) Secondary endpoints: occurrence of SSI, occurrence of urinary tract infection, occurrence of respiratory tract infection, occurrence of other infections, death, occurrence of cardiovascular events, continuous hospitalization on the postoperative30 day, and resistant bacteria in the bacteria causing postoperative SSI, within the postoperative30 day

For each of the secondary endpoints, no statistical test to show the non-inferiority of the short-term group will be conducted by setting a non-inferiority threshold as a null hypothesis. Instead, the risk differences and their 95% confidence intervals will be calculated to assess whether there is an excess risk increase in the short-term group. The risk differences and 95% confidence intervals will be calculated using GEEs in the same manner for the primary endpoint.

3) Subgroup analysis

The primary and secondary endpoints will be analyzed in subgroups defined by institution, time period, or presence/absence of each of the items listed in Section 5.1 (continuous values will be divided by an cutoff specified after data inspection).

4) Interim analysis

No interim analysis will be performed in this study.

7. Registration and Data Collection

7.1. Case Registration

Prior to the start of the study, each institution will be randomly assigned to a short-term or long-term treatment period in advance.

7.2. Participating medical institutions and data registration system

(1) Head of participating medical institution

① Doctors from participating medical institutions who have taken the e-learning course

② Management of access qualifications, etc. of data registrants in the institution.

③ Notify the committee of the facility name, name, medical registration number, and e-mail address, and receive a facility ID and password.

(2) Data registrant

① A physician or medical informaticist from a participating medical institution who has taken the e-learning course, understanding the purpose of this study, and is able to input data honestly and accurately.

② Notify the committee of the facility name, name, medical registration number, and e-mail address, and receive a facility ID and password.

③ ID will be issued to those who meet the above requirements. Only those with an input person ID will be able to enter patient information using a password.

7.3. Data accumulation

Data aggregation is planned to be web-based.

Statistical analysis methods

(1) Target population for analysis: Subjects who meet the eligibility criteria of the study, have started the study treatment after randomization, and for whom data exist. All subjects will be included in the efficacy analysis population. All subjects who have received at least one study treatment will be included in the safety analysis population.

(2) Analysis of the primary efficacy endpoint

(3) Analysis of secondary efficacy endpoints

(4) Analysis of safety endpoints

In the above analysis, the significance level is set at 5% on both sides, unless otherwise noted.

7.3.1. Research period

When the research is terminated, the principal investigator shall report to the head of the research organization in writing without delay to that effect and a summary of the results of the research. The period of the materials and research data retention shall be 5 year after the completion (or discontinuation) of this research or 3 year after the publication of the results, whichever is later. The data materials shall be stored in a locked area or in a storage facility with security measures at the research institution, and shall be destroyed after crushing after the research is completed.

7.3.2. Participating medical institutions

The processing method of research subject IDs will be strictly managed within each facility until the reporting date of research completion. After the completion of the research, the data will be erased or crushed, and then securely destroyed.

7.4. Procedure for modifying and adding registration data

The researcher, etc. should make accurate corrections or additions to the registration data after confirming with the research subject's processing ID.

8. Informed consent

8.1. Explanation to the research subjects

The physician in charge shall prepare and if necessary, revise the explanation and consent documents to obtain consent of the participation from a patient to this research. The prepared or revised documents shall be approved in advance by the Research Ethics Committee. The researcher, etc. shall hand the explanation document which got approved by the research committee in the institution to the research subject before registration, and shall explain the following contents

(Description in the explanatory document)

(1) Permission of the head of the research committee about the name of the research and to perform the study

(2) The name of the research organization and the name of the principal investigator (in cases of collaboration with other research institutions, the name of collaborating institution and the principal investigators shall be included)

(3) Purpose and importnace of the research

(4) Method and time period of the research

(5) Reason for being selected as a research subject

(6) Burden, risks and benefits to the research subjects

(7) A statement that the research subject may withdraw his/her consent at any time even if he/she has given consent to the conduct or continuation of the research (if there is a case where it will be difficult to take measures in accordance with the content of the withdrawal from the research subject, etc., a statement to that effect and the reason).

(8) A statement that the research subject will not be treated disadvantageously by not consenting to the implementation or continuation of the research or by withdrawing consent, and that the research subject will be treated in accordance with standard medical care.

(9) Method of disclosing information about the research

(10) The fact that the research protocol and materials related to the research methods may be obtained or inspected at the request of the research subjects, etc., to the extent that it does not interfere with the protection of the personal information of other research subjects, etc., and the securing of originality of the research, and the method of obtaining or inspecting such materials.

(11) Handling of personal information, etc. (including process of anonymization, when anonymization is conducted)

(12) Method of storage and disposal of samples and information

(13) Status of research-related conflicts of interest of the research implementing entity, such as research fund resources, as well as research-related conflicts of interest of each investigator, etc., such as his/her individual income;

(14) Response to inquiries, etc. from research subjects and related parties

(15) If there is any financial burden or gratuity to the research subjects, etc., a statement to that effect and the details thereof.

(16) As this research does not involve any medical treatment beyond normal medical care, even if the subject does not consent to this research, we will provide treatment in accordance with standard medical care.

(17) As this research does not involve medical treatment beyond the scope of normal medical care, questions regarding the provision of medical care to research subjects after the research has been conducted will be accepted on an individual basis.

(18) When the implementation of the research may result in important findings concerning the health of the research subject, genetic characteristics that may be passed on to offspring, etc., the results of the research pertaining to the research subject (including incidental findings) shall be handled in a manner that is appropriate for the research. Handling of research results pertaining to research subjects including incidental findings.

(19) If there is a possibility that samples/information obtained from research subjects will be used for future research that is not specified at the time consent is obtained from the research subjects, etc., or will be provided to other research institutions, a statement to that effect and the details expected at the time consent is obtained.

8.2. Agreement

1) Before the patient participates in this research, the physician in charge shall obtain the patient's free and voluntary consent to participate in this research from the patient by means of a written consent form, after providing the patient with sufficient explanation using an explanation document approved by the Research Ethics Committee.
2) The physician in charge must give the patient an opportunity to ask questions and sufficient time for the research subject or others to refuse before consent is obtained. At that time, the physician in charge or the research collaborator as a supplementary explanator must answer all questions to the satisfaction of the patient.
3) The consent document should be signed and dated by the physician in charge who provided the explanation and the patient. If supplementary explanations are given by a research collaborator, the research collaborator should also sign and date the document.
4) The physician in charge will provide the patient with a signed and dated copy of the consent document and an explanation document before the patient participates in the study.

5) When information that may affect the patient's decision on whether or not to participate in the research is obtained, the physician in charge shall promptly inform the patient of such information, confirm the patient's decision on whether or not to continue to participate in the research, and record the fact that such information has been informed to the patient together with the date in the medical record.

9. Publication of research results

Prior to the start of subject enrollment, the study plan will be registered with the University Hospital Medical Information Network: UMIN or clinicaltrails.gov (http://www.clinicaltrials.gov). The results will be presented at conferences targeting surgical site infections as appropriate, and will be submitted to general medical clinical journals not limited to bone and joint journals.

10. Personal Information, etc.

10.1. Purpose of use of personal information

In order to obtain the correct results of the research, personal information will be used for the purpose of identifying and surveying individual research subjects at least 30 day (within 180 days) after the surgery, and for the purpose of appropriately managing the information obtained.

10.2. Method of use (method of anonymization)

The personal information of research subjects will be managed at the data registrar as both an anonymized processed research subject ID ① consisting of the institution number and registration sequential number of the institution concerned, and another research subject ID② in which medical record numbers, etc. are anonymized and further processed according to the institutional rules of each participating cooperative institution.

Only research subject IDs ① will be disclosed from the data registries to the research secretariat, and research subject IDs ② and other information that may identify individuals will not be disclosed from the data registries to the research secretariat. The method of creating research subject IDs ② will be strictly controlled within each data registration institution and will not be disclosed.

In addition, if a separate table of correspondence with the research subject ID ① is created within the data registration institution, it shall be strictly managed under the responsibility of the head of the data registration institution.

10.3. Safety management responsibility system (safety management measures for personal information)

The basic principle is that personal information, etc. obtained in the course of conducting research does not belong to individual researchers, etc., but is held by each data registration institution and is subject to necessary and appropriate management and supervision. For this reason, researchers, etc. must not disclose personal information held by the data registrar to which they belong (including cases where such information is entrusted

to be stored). For this reason, researchers, etc. shall not leak, lose, or destroy personal information held by the data registrar to which they belong (including personal information stored by entrustment; hereinafter referred to as "retained personal information, etc."). In addition, researchers, etc. must appropriately handle personal information, etc. (hereinafter referred to as "personal information, etc.") held by the data registries to which they belong in order to prevent leakage, loss, or damage and for other safety management purposes. These shall be thoroughly communicated to all researchers and others through e-learning at the time of participation in the research.

In addition, the management of this registration system has been entrusted to the following company that has obtained the Privacy Mark (P Mark) for safety management. Although this research does not handle information that can easily identify individuals (name, date of birth, etc.), it is strictly managed in the same way as personal information in accordance with the Privacy Mark standards. The server is located at NTT PC Communications Corporation (Chiyoda Ward, Tokyo), and strong security is applied to prevent outsiders from obtaining the information, and the address is not disclosed.

First Corporation (P Mark registration number:17001439)
Shimizu Building 4F, 8-14-18 Nishi-Shinjuku, Shinjuku-ku, Tokyo 160-0023, Japan
TEL: 03-5332-6644 FAX: 03-5332-6651

11. Proper handling of personal information etc.

11.1 Storage

11.1.1 Storage at research institutions

The web-registered research data will be stored on the server of NTT PC Communications, Inc. under the responsibility of the research secretariat. The data will be stored for 5 years after the completion (or discontinuation) of this study.

11.1.2 Storage at data registries

In the data registration period, research subject IDs ① and ② and registration data will be stored. The storage period shall be three years from the date of provision.

11.2 Disposal

11.2.1 Disposal at research institutions

The research data registered on the web will be deleted from the server of NTT PC Communications, Inc. at the end of the storage period under the responsibility of the research secretariat.

11.2.2 Disposal at the data registrar

Research subject IDs (1), (2) and registration data will be destroyed after the storage period.

12. Burden on the research subjects, anticipated risks (including possible adverse events) and benefits, and these Comprehensive evaluation, measures to minimize burden and risk

12.1. Summary of anticipated benefits and disadvantages associated with participation in the study

The antimicrobial agents to be used in this study are those recommended by global guidelines, and the duration of administration is also that recommended by national guidelines. We believe that the safety of the administration method is within the standard range of use and that there are no major problems.

As a common side effect of antimicrobial agents, anaphylactic shock may occur in extremely rare cases, so thorough investigation of the symptoms should be conducted, and if it occurs, appropriate medical examination and treatment should be provided immediately.

12.2. Response to Disadvantages Arising from Participation in Research

(1) Compensation for health damage

In the event that a subject suffers health damage as a result of participating in this study, the implementing medical institution will take necessary and appropriate measures, including providing a medical system for the treatment of the damage. In preparation for compensation liability, this study will be covered by clinical research insurance.

(2) Obtaining liability insurance

The principal investigator and sub-investigators will be covered by liability insurance.

13. Status of conflicts of interest related to research of researchers, etc., including sources of funding for research, etc., conflicts of interest related to research of research institutions and earnings of individuals, etc.

This is an academia-led study mainly supported by the Tokyo Metropolitan Government, the Japan Labor Health and Welfare Organization, and the Grant-in-Aid for Scientific Research (Grant-in-Aid for Scientific Research Foundation/Subsidy for Scientific Research). There are no conflicts of interest related to this research topic at all participating sites. This study will be initiated after obtaining the approval of the Ethics Committee at each participating institution.

14. Intellectual Property

The results, data, and intellectual property rights obtained from this research belong to the OSSI Secretariat. The specific handling and allocation of intellectual property rights will be determined through consultation. Whether the intellectual property of the principal investigator, Yuji Okazaki, is to be attributed to an individual or to a research institution is subject to the agreement of the research institution to which he belongs.

15. Public announcement

15.1. Registration of research plan

The principal investigator will register the research outline in the public database (University Hospital Medical Information Network (UMIN) (http://www.umin.ac.jp/ctr/index-j.htm)) and update it as appropriate according to changes in the research protocol and research progress.

15.2. Registration of Research Results

The principal investigator will register the results of the research in public databases, etc., after the research is completed. However, the human rights of research subjects, the human rights of researchers and other related parties, matters that are not disclosed for the protection of intellectual property, and matters that are approved by the head of the research organization after receiving the opinion of the Ethics Committee because they would significantly interfere with the research from the perspective of protecting personal information shall not be disclosed.

15.3. Publication of research results

After the completion of the research, the principal investigator will publish the research results in medical journals without delay, after taking measures to protect the personal information of the research subjects.

When the final publication of the results is made, it shall be reported to the head of the research institution without delay.

16. Method of reporting to the head of the research organization

The principal investigator shall report the following to the head of the research institution

- Progress of the research
- Occurrence of adverse events associated with the implementation of the research
- End of study/discontinued, summary of results

17. Response to consultations, etc., from research subjects, etc., and their related persons

The following is the contact point for inquiries about research in general.

<Representatives of each facility>

*Yasuhito Tajiri, Department of Orthopaedic Surgery, Tokyo Metropolitan Hiroo Hospital

*Hiroshi Okazaki, Department of Orthopedics, Kanto Rosai Hospital
*Kiyofumi Yamakawa, Department of Orthopaedic Surgery, Tokyo Metropolitan Komagome Hospital
*Takuya Matsumoto, Department of Orthopaedic Surgery, Tokyo Metropolitan Tama General Hospital
*Fumiaki Tokimura, Tokyo Metropolitan Institute of Gerontology and Gerontology
*Koji Yamada Research Office

18. Financial burdens or rewards for the research subjects

This study is a treatment within the scope of insurance coverage, and no special additional patient cost burden will be incurred. In addition, confirmation of the postoperative course at the postoperative 30date and time is a normal part of medical treatment, and is not a special treatment. In view of the above, we believe that payment of the burden reduction fee for participation in this study is unnecessary.

19. Handling of research results (including incidental findings) pertaining to research subjects when there is a possibility of important insights into the health of the research subjects, genetic characteristics that can be passed on to their offspring

Communicate any important findings regarding the health of the research subjects.

20. Method of supervision for description of business and contractors

Data analysis will be entrusted to the person in charge of statistical analysis. System construction, system management, and server management of the database will be outsourced to First Corporation, and the principal investigator and the statistician will participate in the OSSI research group to share information and supervise the operation.

21. The possibility that the information will be used for future research that is not identified at the time the consent is received. / Possibility of the information to be provided to other research institutions

Of the data obtained in this research, those for which written consent has been obtained in advance for secondary use in medical research to be newly planned and conducted in the future will be used after approval by the Ethics Committee.

22. Provision of samples and information, etc., to research institutions collaborating in this research or other research institutions

Information will be managed by the research secretariat and will not be provided to other institutions unless approved by the OSSI research group and approved by the ethics committee, except for what will be published in ".15.3 Publication of Research Results".

23. Provision of samples, information, etc. to commercial organizations, etc.

Information will be managed by the research office and will not be provided to other commercial or private organizations unless approved by the OSSI research group and approved by the ethics committee, except for what will be published in "15.3 Publication of Research Results.".

24. Response to adverse events

(1) Response to subjects in the event of an adverse event

An adverse event is defined as any unfavorable medical event that occurs in a subject up to 30 days after surgery. When an adverse event is recognized, the investigator or sub-investigator should immediately take appropriate measures and document the event in the medical record and case report without discrepancy. If the administration of the study drug is discontinued or treatment for the adverse event becomes necessary, the subject should be informed. In the case report form, the name of the adverse event, date of occurrence, severity, status of intervention by the study, outcome, and causal relationship to the drug used in this study should be recorded. The adverse events expected in this study will be defined by the side effects described in the drug package insert.

Criterion of causal relationships

① Relationships can be denied: No correlation in time, or when the event was considered due to other factors such as primary disease, complications, concomitant medication, concomitant treatment, etc.

② Relationships cannot be denied: If the above definition of "relationships can be denied" is not met (including cases of insufficient evaluation materials)

(2) Reporting of serious adverse events

① Serious adverse events are defined as Table 2.

② If an adverse event that occurs is judged to be serious, the principal investigator will promptly report it to the head of the medical institution where the research is being conducted (e.g., the ethics committee of each institution) and enter it into the SAE report form on the EDC. The research secretariat will support the preparation of adverse event reports based on the ethical guidelines for clinical research and the work of sharing information with the study participating institutions. Details of the procedure will follow the Safety Information Management SOP of the Clinical Research Support Center, University of Tokyo Hospital.

③ In the event of an unexpected serious adverse event or malfunction related to clinical research that is

invasive, the principal investigator shall promptly report it to the head of the medical institution where the research is being conducted (institutional ethics committee, etc.), and shall cooperate with the head of the medical institution where the research is being conducted in reporting it to the Minister of Health, Labor and Welfare and making it public. In addition, we will cooperate with the head of the research institution in reporting and publicizing the results to the Minister of Health, Labor and Welfare.

④ Post-marketing drugs should be reported to the Ministry of Health, Labour and Welfare (MHLW) through the MHLW's Drug Safety Information Reporting System.

Table 2: Serious adverse events

1	Death
2	Cases that may lead to death
3	Cases that require hospitalization or extended hospitalization at a hospital or clinic for treatment.
4	Handicapped
5	Cases that may lead to handicap
6	Severe cases regarding above 1-5
7	Congenital diseases or abnormalities that may affect later generations

25. Handling of deviations from the initial plan

Compliance with the research protocol

This research will be conducted under the agreement between the physician in charge and the principal investigator, and in compliance with this research protocol.

Deviations from the research protocol

The physician in charge may make deviations or changes from the research protocol without prior written agreement with the principal investigator and prior approval of the research ethics committee due to unavoidable medical circumstances in order to avoid disadvantages to patients. In such cases, the physician in charge will prepare a record explaining the details of the deviation and the reasons for it, and submit it to the head of the medical institution where the research is being conducted and the research secretariat as necessary.

26. Changes to the research protocol

① If the need to revise the research protocol arises during the conduct of this research, the physician in charge shall consult with the principal investigator to determine the details of the revision.

② The principal investigator will promptly report the revision and the reason for the revision in writing to the physician in charge. The physician in charge and the principal investigator will consult and agree on the revised research protocol.

③ The physician in charge submits a revised version of the research protocol agreed upon with the principal investigator to the head of the medical institution where the research is to be conducted, and obtains the approval of the Ethics Committee if necessary.

27. Research organization

Principal Investigator.

Kanto Rosai Hospital, Department of Orthopedics and Spine Surgery Tel: 0444113131 (Hospital Representative)

Hiroshi Okazaki, Chief, Division of Orthopaedic and Spine Surgery, Deputy Director (Principal Investigator)

Department of Orthopedics, Tokyo Metropolitan Hiroo Hospital Tel: 0334441181 (Hospital Representative)

Yasuhito Tajiri, Director, Department of Orthopaedic Surgery, Deputy Director (Co-Principal Investigator)

Research Secretariat

Department of Orthopedics and Spine Surgery, Kanto Rosai Hospital

Orthopaedic Surgical Site Infection (OSSI) Study Group Secretariat Tel:0444113131 (Hospital Representative)

Koji Yamada, Chief, Department of Orthopedics and Spine Surgery

The University of Tokyo Hospital Tel: 0338155411 (Hospital Representative)

Kosei Nagata, Orthopedic Surgery, Spine Surgery

Head of Statistical Analysis

Department of Medical Research and Management for Musculoskeletal Pain, Graduate School of Medicine, The University of Tokyo

Hiroyuki Oka, Specially Appointed Associate Professor

Division of Biostatistics, Department of Health Science and Nursing, Graduate School of Medicine, The University of Tokyo

Tomohiro Shinozaki, Assistant Professor

7-3-1, Hongo, Bunkyo-ku, Tokyo 113-0033 , Japan TEL : 03-3815-5411
