# Supplementary material for: Effect of Antimicrobial Prophylaxis Duration on Health Care–Associated Infections After Clean Orthopedic Surgery: A Cluster Randomized Trial
Source: JAMA Netw Open. 2022 Apr 12;5(4):e226095. doi: 10.1001/jamanetworkopen.2022.6095 (PMC9006110; doi:10.1001/jamanetworkopen.2022.6095)
Supplement: Supplement 2. — eTable 1. Unified Surgical Site Infection Prevention Protocols eTable 2. Detailed Information of Patients With Healthcare-Associated Infections eTable 3. Primary and Secondary Endpoints of the Culture Positive Patients in the Intention-to-Treat Population eTable 4. Detailed Information of Patients Who Readmitted the Hospital Within 30 Days After Surgery eFigure 1. Time Course of Participants’ Recruitment eFigure 2. Time Course of Follow-up Rate During the 30−180 Days After Surgery eFigure 3. Forest Plot of Risk Ratios (95% Confidence Interval [CI]) for Healthcare-Associated Infections by Subgroups (Group 24 Versus Group 48) eFigure 4. Forest Plot of Risk Ratios (95% Confidence Interval [CI]) for Surgical Site Infections by Subgroups (Group 24 Versus Group 48) eFigure 5. Probability of Hospital Stays in Group 24 and Group 48 During 30 Days After Surgery [file jamanetwopen-e226095-s002.pdf]

Supplemental Online Content

Nagata K, Yamada K, Shinozaki T, et al; OSSI investigators. Effect of antimicrobial prophylaxis duration on health care–associated infections after clean orthopedic surgery: a cluster randomized trial. *JAMA Netw Open.* 2022;5(4):e226095. doi:10.1001/jamanetworkopen.2022.6095

eTable 1. Unified Surgical Site Infection Prevention Protocols

eTable 2. Detailed Information of Patients With Healthcare-Associated Infections

eTable 3. Primary and Secondary Endpoints of the Culture Positive Patients in the Intention-to-Treat Population

eTable 4. Detailed Information of Patients Who Readmitted the Hospital Within 30 Days After Surgery

eFigure 1. Time Course of Participants' Recruitment

eFigure 2. Time Course of Follow-up Rate During the 30−180 Days After Surgery

eFigure 3. Forest Plot of Risk Ratios (95% Confidence Interval [CI]) for Healthcare-Associated Infections by Subgroups (Group 24 Versus Group 48)

eFigure 4. Forest Plot of Risk Ratios (95% Confidence Interval [CI]) for Surgical Site Infections by Subgroups (Group 24 Versus Group 48)

eFigure 5. Probability of Hospital Stays in Group 24 and Group 48 During 30 Days After Surgery

This supplementary material is provided by the authors to provide the readers with additional information on their work.

eTable 1.Unified surgical site infection prevention protocols

(1) Preoperative shaving with electrical clippers immediately before skin incision, when necessary
(2) Preoperative skin preparation at least twice with povidone iodine with or without alcohol before using adhesive drapes
(3) Double gloving with outer glove exchange at least every 2 hours for all operating staff member
(4) Using iodine-impregnated adhesive drapes after skin preparation in all procedures except for hand or foot surgeries
(5) Keeping operation room traffic to a minimum
(6) Using a laminar air flow-equipped theatre for total arthroplasty
(7) Administering initial antimicrobial prophylaxis before surgery with completion before tourniquet inflation
(8) Maintaining normothermia by forced-air warming systems
(9) Using antimicrobial-coated sutures for closure
(10) Avoiding administration of local antibiotics, including antibiotic-impregnated cement or vancomycin powder

To minimize the effect of confounding factors, all participating institutions agreed to perform the following SSI prevention protocols shown above. The staff members of the participating hospitals were thoroughly informed to comply with these steps, including (1), (3), (5), and (6). The participating doctors logged to ensure achievement of these steps, including (2), (4), (7), (8), (9), and (10).
SSI, Surgical site infection; AMP, antimicrobial prophylaxis

© 2022 Nagata K et al. *JAMA Network Open.*

eTable2. Detailed information of patients with healthcare-associated infections

Group	Age (years)	Sex	ASA	Surgery	Type of HAI	Onset*	HAI Pathogens
Group 24	50	M	2	Spine with instrumentation	Deep SSI	17	*Staphylococcus, coagulase - negative*
Group 24	75	F	2	Arthroplasty	Deep SSI[c]	7	Culture negative
Group 24	64	F	2	Spine with instrumentation	Deep SSI<br>UTI	16<br>18	MSSA<br>*Enterococcus faecalis*
Group 24	56	M	3	Spine with instrumentation	Deep SSI[c]<br>UTI	9<br>9	*Staphylococcus epidermidis*<br>*Escherichia coli*
Group 24	93	F	2	Open reduction internal fixation	SSI	7	*Staphylococcus epidermidis*
Group 24	61	M	2	Spine without instrumentation	SSI	23	Culture negative
Group 24	59	F	2	Spine with instrumentation	SSI	6	No culture obtained
Group 24	59	M	2	Spine without instrumentation	SSI	26	No culture obtained
Group 24	84	F	2	Arthroplasty	SSI	12	No culture obtained
Group 24	79	F	2	Arthroplasty	SSI	11	No culture obtained
Group 24	91	M	2	Spine without instrumentation	SSI	12	Culture negative
Group 24	82	F	2	Arthroplasty	SSI	16	No culture obtained
Group 24	74	M	2	Spine with instrumentation	SSI	25	Culture negative
Group 24	83	F	2	Arthroplasty	SSI	15	MSSA
Group 24	95	M	3	Open reduction internal fixation	UTI	7	ESBL-producing *Escherichia coli*
Group 24	77	F	2	Spine with instrumentation	UTI[d]	6	ESBL-producing *Escherichia coli*
Group 24	86	F	2	Open reduction internal fixation	UTI	10	Culture negative
Group 24	74	F	2	Arthroplasty	UTI	14	*Escherichia coli*
Group 24	76	M	2	Spine without instrumentation	UTI	11	Culture negative
Group 24	75	F	2	Spine without instrumentation	UTI	7	*Escherichia coli, Enterococcus faecalis, Staphylococcus lugdunensis*
Group 24	79	M	2	Spine with instrumentation	UTI	6	*Enterobacter cloacae*
Group 24	86	F	2	Arthroplasty	UTI	8	*Escherichia coli*
Group 24	91	F	3	Open reduction internal fixation	UTI	28	*Escherichia coli*
Group 24	84	M	2	Open reduction internal fixation	RTI	2	Culture negative
Group 24	86	M	3	Spine without instrumentation	RTI	4	Culture negative

© 2022 Nagata K et al. *JAMA Network Open.*

Group 24[a]	82	M	3	Open reduction internal fixation	RTI	17	*Escherichia coli*
Group 24	81	F	2	Arthroplasty	Cholecystitis	25	Culture negative
Group 24	74	M	2	Spine without instrumentation	Oral ulcer and pulpitis	14	No culture obtained
Group 24	54	M	2	Arthroplasty	Cellulitis	10	No culture obtained

eTable2. Continued

Group	Age (years)	Sex	ASA	Surgery	Type of HAI	Onset*	HAI Pathogens
Group 48	54	F	2	Arthroplasty	Deep SSI	14	*Staphylococcus, coagulase negative*
Group 48	70	M	1	Spine with instrumentation	Deep SSI	12	*Escherichia coli*
Group 48	82	M	3	Spine with instrumentation	Deep SSI	20	*Staphylococcus lugdunensis*
Group 48	65	M	2	Spine with instrumentation	Deep SSI	12	MSSA
Group 48	70	M	2	Spine with instrumentation	Deep SSI	18	MRSA
Group 48	60	M	3	Spine with instrumentation	Deep SSI	14	*Staphylococcus haemolyticus*
Group 48	76	F	2	Arthroplasty	Deep SSI	19	MSSA
Group 48	54	F	2	Open reduction internal fixation	SSI	21	No culture obtained
Group 48	83	F	2	Arthroplasty	SSI	13	Culture negative
Group 48	83	F	2	Open reduction internal fixation	SSI	25	No culture obtained
Group 48	68	M	2	Arthroplasty	SSI	18	No culture obtained
Group 48	75	M	2	Arthroplasty	SSI	14	Culture negative
Group 48	77	F	2	Arthroplasty	SSI	30	No culture obtained
Group 48	60	F	2	Arthroplasty	SSI	11	MRSA
Group 48	73	M	2	Nerve transfusion	SSI	8	Culture negative
Group 48	79	M	2	Spine with instrumentation	SSI	10	*Pseudomonas aeruginosa*
Group 48	68	F	2	Arthroplasty	SSI	18	MSSA, *Enterobacter cloacae*
Group 48	71	M	2	Arthroplasty	SSI	7	Culture negative
Group 48[b]	67	F	1	Open reduction internal fixation	SSI	22	*Bacillus* spp.
Group 48	85	M	2	Spine with instrumentation	UTI<br>CRBSI	7<br>15	*Citrobacter* spp.<br>*Acinetobacter baumannii* (anitratus)
Group 48	78	M	3	Arthroplasty	UTI	26	Culture negative
Group 48	77	F	2	Spine with instrumentation	UTI	5	*Proteus* spp.
Group 48	56	M	2	Spine without instrumentation	UTI	7	*Klebsiella pneumoniae, Escherichia coli*

© 2022 Nagata K et al. *JAMA Network Open.*

Group 48	80	F	2	Spine with instrumentation	UTI	13	Culture negative
Group 48	83	F	2	Spine with instrumentation	UTI[d]	21	*Escherichia coli*
Group 48	87	F	3	Open reduction internal fixation	UTI	30	Culture negative
Group 48	53	M	2	Spine with instrumentation	UTI	7	ESBL-producing *Escherichia coli*
Group 48	49	M	1	Open reduction internal fixation	UTI	12	*Escherichia coli*
Group 48	91	F	2	Arthroplasty	UTI	3	ESBL-producing *Escherichia coli, Streptococcus agalactiae*
Group 48	81	M	2	Spine with instrumentation	UTI	9	*Enterobacter* spp.
Group 48	85	F	2	Arthroplasty	UTI	1	γ-*Streptococcus*
Group 48	79	F	2	Arthroplasty	UTI	15	Culture negative
Group 48	80	F	2	Arthroplasty	RTI	11	Culture negative

eTable2. continued

Group	Age (years)	Sex	ASA	Surgery	Type of HAI	Onset*	HAI Pathogens
Group 48	82	M	2	Spine with instrumentation	RTI	7	Culture negative
Group 48	93	F	2	Arthroplasty	RTI	18	*Chryseomonas* spp., *Neisseria* spp.
Group 48	85	M	2	Open reduction internal fixation	RTI	3	*Enterobacter* spp., *Pseudomonas aeruginosa, Klebsiella pneumoniae*
Group 48	81	M	3	Spine without instrumentation	RTI	7	No culture obtained
Group 48	77	M	2	Spine without instrumentation	unknown fever	5	No culture obtained

Abbreviations: HAI, healthcare-associated infection; ASA, American Society of Anesthesiologists physical status; SSI, surgical site infection; UTI, urinary tract infection; RTI, respiratory tract infection; CRBSI, catheter-related blood stream infection; MSSA, methicillin-sensitive *Staphylococcus aureus*; MRSA, methicillin-resistant *Staphylococcus aureus*; ESBL, extended-spectrum beta-lactamase.

*: Onset days after surgery.

a: Allocated to Group 24 but antimicrobial prophylaxis was administrated for 48 hours after surgery.

b: Allocated to Group 48 but antimicrobial prophylaxis was administrated for more than 48 hours after surgery.

c: Required surgical debridement.

d: Readmitted to the hospital for non-HAI reasons and experienced UTI in the second admission within 30 days after surgery.

© 2022 Nagata K et al. *JAMA Network Open.*

eTable 3. Primary and secondary endpoints of the culture positive patients in the intention-to-treat population

Endpoint	Group 24 (n=633) Event— n (%)	Group 48 (n=578) Event— n (%)	Risk difference (%) (95% CI) [a]	Risk ratio (%) (95% CI) [a]
Primary				
HAI	12 (1.9)	23 (4.0)	-2.08 (-4.38 to 0.22)	0.48 (0.22 to 1.03)
Secondary				
SSI	5 (0.8)	11 (1.9)	-1.11 (-2.29 to 0.06)	0.42 (0.17 to 1.01)
UTI	9 (1.4)	9 (1.6)	-0.14 (-1.86 to 1.59)	0.91 (0.29 to 2.87)
RTI	0 (0)	3 (0.5)	-0.52 (-1.14 to 0.10)	N/A
Other infectious diseases	0 (0)	1 (0.2)	-0.17 (-0.44 to 0.09)	N/A
Deep SSI	4 (0.6)	7 (1.2)	-1.74 (-4.34 to 0.87)	0.52 (0.18 to 1.52)

All outcomes were dichotomized into presence or absence at patient-level.
a: Estimated by linear- and log-linear-risk generalized estimating equations (with canonical link) clustering hospitals and periods.
AMP, antimicrobial prophylaxis; CI, confidence interval; HAI, healthcare-associated infection; N/A, not applicable; SSI, surgical site infection; UTI, urinary tract infection; RTI, respiratory tract infection.

© 2022 Nagata K et al. *JAMA Network Open.*

eTable 4. Detailed information of patients who readmitted the hospital within 30 days after surgery

Group	Age (years)	Sex	Surgery	Discharge[a]	Readmission[a]	Cause of readmission
Group 24	68	M	Open reduction internal fixation	17	23	Diverticulitis
Group 24[b]	77	F	Spine with instrumentation	15	17	Hyponatremia
Group 24	77	F	Spine with instrumentation	21	26	Vertebral cage migration after L4/5 TLIF
Group 48	56	M	Spine with instrumentation	14	25	Late cerebrospinal fluid leakage
Group 48	57	M	Open reduction internal fixation	26	30	Seizure due to brain metastasis of lung cancer
Group 48[b]	83	F	Spine with instrumentation	12	13	Sacral fracture due to falling
Group 48	86	F	Open reduction internal fixation	18	25	Contralateral femoral neck fracture due to falling

a: Days after primary surgery to discharge or readmission.

b: These two patients suffered urinary tract infection after readmission described in eTable 2.

TLIF, transforaminal interbody fusion

© 2022 Nagata K et al. *JAMA Network Open.*

eFigure 1. Time course of participants' recruitment

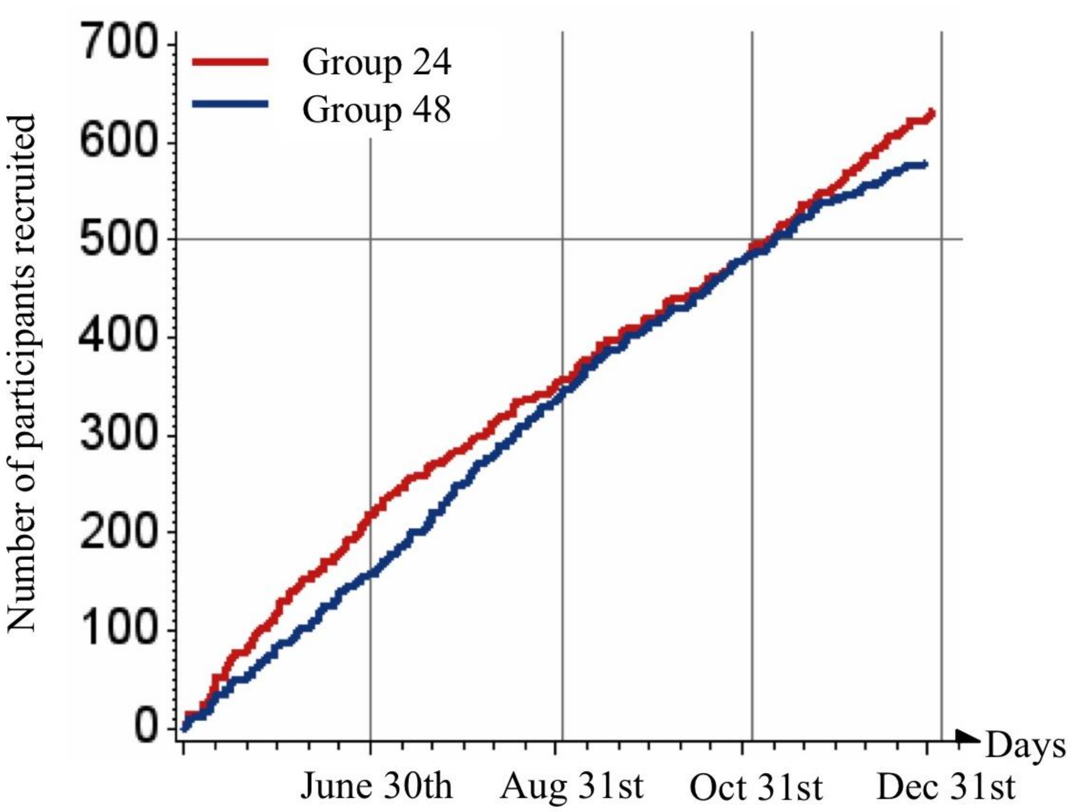

Number of patients recruited

	June 30th	Aug 31st	Oct 31st	Dec 31st
Group 24	219	357	494	633
Group 48	157	346	486	578

© 2022 Nagata K et al. *JAMA Network Open.*

eFigure 2. Time course of follow-up rate during the 30−180 days after surgery

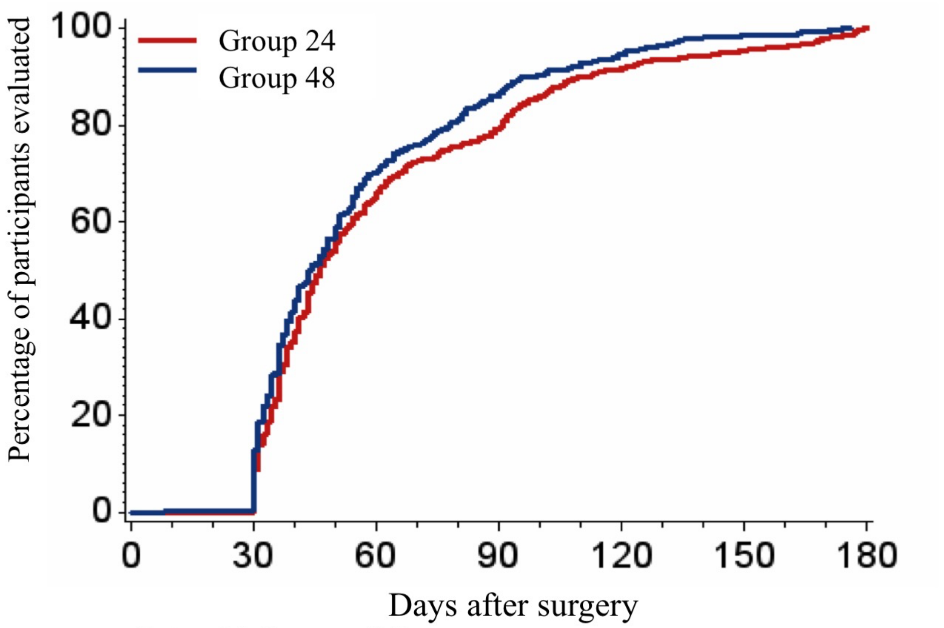

Rate of follow-up (%)

	Day 60	Day 90	Day 120	Day 150	Day 180
Group 24	66.0	79.6	91.8	95.4	100
Group 48	70.6	86.7	94.6	98.4	100

© 2022 Nagata K et al. *JAMA Network Open.*

eFigure 3. Forest plot of risk ratios (95% confidence interval [CI]) for healthcare-associated infections by subgroups (Group 24 versus Group 48)

Subgroup	Group 24 n	Group 24 HAI	Group 24 %	Group 48 n	Group 48 HAI	Group 48 %	Risk ratio	(95% CI)
Age <65 years	197	7	3.6	158	7	4.4	0.80	(0.21, 3.09)
Age 65-<80 years	265	9	3.4	258	16	6.2	0.55	(0.23, 1.33)
Age ≤80 years	171	13	7.6	162	15	9.3	0.82	(0.40, 1.67)
Sex, Male	250	14	5.6	204	20	9.8	0.57	(0.29 1.14)
Sex, Female	383	15	3.9	374	18	4.8	0.81	(0.38, 1.75)
Diabetes	72	8	11.1	59	7	11.9	0.94	(0.40, 2.18)
No diabetes	556	21	3.8	512	31	6.1	0.62	(0.33, 1.18)
Hemodialysis	5	0	0	4	0	0	NA	
No hemodialysis	628	29	4.6	574	38	6.6	0.70	(0.38, 1.49)
Rheumatoid arthritis	27	0	0	32	1	3.1	NA	
No rheumatoid arthritis	606	29	4.8	546	37	6.8	0.71	(0.39, 1.28)
BMI <25 kg/m2	415	16	3.9	381	23	6.0	0.64	(0.31 1.30)
BMI ≥25 kg/m2	218	13	6.0	197	15	7.6	0.78	(0.39, 1.57)
ASA 1 or 2	570	24	4.2	523	33	6.3	0.67	(0.35,1.26)
ASA 3 or 4	63	5	7.9	55	5	9.1	0.87	(0.37, 2.08)
Open reduction internal fixation	119	6	5.0	117	6	5.1	0.98	(0.35, 2.77)
Arthroplasty	273	9	3.3	245	15	6.1	0.54	(0.21, 1.40)
Arthrodesis	6	0	0	3	0	0	NA	
Osteotomy	6	0	0	2	0	0	NA	
Spine without instrumentation	108	7	6.5	98	3	3.1	2.12	(0.60, 7.41)
Spine with instrumentation	94	7	7.4	93	13	14.0	0.53	(0.18, 1.55)
Arthroscopy	10	0	0	9	0	0	NA	
Bone tumor resection	0	0	0	1	0	0	NA	
Soft tissue tumor resection	3	0	0	3	0	0	NA	
Other	14	0	0	7	1	14.3	NA	
No urinary catheter	84	0	0	46	2	4.3	NA	
Urinary catheter <24 h	159	5	3.1	175	10	5.7	0.55	(0.19, 1.59)
Urinary catheter ≥24 h	390	24	6.2	357	26	7.3	0.84	(0.46, 1.55)
Surgical drain closed, <48 h	296	10	3.4	256	16	6.3	0.54	(0.19, 1.51)
Surgical drain closed, ≥48 h	258	14	5.4	253	15	5.9	0.92	(0.51,1.63)
Surgical drain none	67	5	7.5	66	7	10.6	0.70	(0.27, 1.81)
Surgical drain not closed, <48 h	12	0	0	3	0	0	NA	
Surgical drain not closed, ≥48 h	12	0	0	3	0	0	NA	
Hospital 1	310	15	4.8	263	21	8.0	0.61	(0.42, 0.88)
Hospital 2	111	0	0	79	1	1.3	NA	
Hospital 3	56	4	7.1	56	4	7.1	1.00	(0.21, 4.73)
Hospital 4	48	1	2.1	66	3	4.5	0.46	(0.19, 1.08)
Hospital 5	108	9	8.3	114	9	7.9	1.06	(0.34, 3.32)
May–June 2018	219	8	3.7	157	11	7.0	0.52	(0.26, 1.05)
July–August 2018	138	12	8.7	189	15	7.9	1.10	(0.41, 2.92)
Sept-Oct 2018	137	1	0.7	140	9	6.4	0.11	(0.01, 0.54)
Nov-Dec 2018	139	8	5.8	92	3	3.3	1.76	(0.91, 3.42)

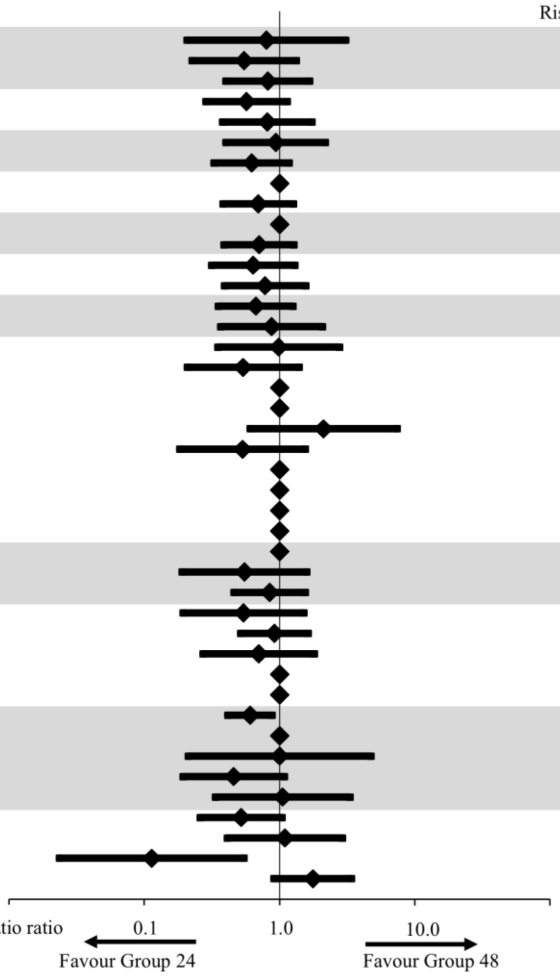

© 2022 Nagata K et al. *JAMA Network Open.*

eFigure 3. continued.

Subgroup	Group 24 n	Group 24 HAI	Group 24 %	Group 48 n	Group 48 HAI	Group 48 %	Risk ratio (95% CI)	
Smoker	69	3	4.3	62	2	3.2	1.35	(0.37, 4.94)
Non-smoker	564	26	4.6	516	36	7.0	0.66	(0.34, 1.28)
Operative time <150 min	435	17	3.9	386	14	3.6	1.08	(0.51, 2.28)
Operative time ≥150 min	198	12	6.1	192	24	12.5	0.48	(0.23, 1.00)
Estimated blood loss <1000 mL	618	28	4.5	559	36	6.4	0.70	(0.40, 1.24)
Estimated blood loss ≥1000 mL	15	1	6.7	19	2	10.5	0.63	(0.07, 5.92)
General anaesthesia	502	21	4.2	448	29	6.5	0.65	(0.34, 1.23)
No general anaesthesia	131	8	6.1	130	9	6.9	0.88	(0.44,1.76)
Spinal anaesthesia	130	8	6.2	134	9	6.7	0.92	(0.45, 1.88)
No spinal anaesthesia	503	21	4.2	444	29	6.5	0.64	(0.34, 1.21)
Epidural anaesthesia	83	4	4.8	86	0	0	NA	
No epidural anaesthesia	550	25	4.5	492	38	7.7	0.59	(0.28, 1.25)
Peripheral block	29	1	3.4	23	3	13.0	0.26	(0.05, 1.36)
No peripheral block	604	28	4.6	555	35	6.3	0.74	(0.41, 1.32)
Emergency surgery	21	2	9.5	22	3	13.6	0.70	(0.10, 4.65)
Scheduled surgery	612	27	4.4	556	35	6.3	0.70	(0.38, 1.29)
Revision surgery	25	2	8.0	38	7	18.4	0.43	(0.15, 1.22)
Primary surgery	608	27	4.4	540	31	5.7	0.77	(0.40, 1.48)
CEZ administration	623	29	4.7	575	38	6.6	0.70	(0.39, 1.28)
no CEZ administration (VCM or CLDM only)	10	0	0	3	0	0	NA	
Nasal decolonisation	40	5	12.5	54	11	20.4	0.61	(0.41, 0.92)
No nasal decolonisation	593	24	4.0	524	27	5.2	0.79	(0.38, 1.62)
Skin preparation by Iodine-alcohol	400	16	4.0	343	20	5.8	0.69	(0.36, 1.32)
Skin preparation by Iodine	228	13	5.7	225	18	8.0	0.71	(0.28, 1.80)
Skin preparation by chlorhexidine alcohol	4	0	0	9	0	0	NA	
Skin preparation by 0.5% chlorhexidine gluconate	0	0	0	1	0	0	NA	
Skin preparation by olanexidine gluconate	1	0	0	0	0	0	NA	
Iodine drape	587	29	4.9	546	38	7.0	0.71	(0.39, 1.28)
No iodine drape	46	0	0	32	0	0	NA	
Iodine irrigation	187	14	7.5	204	24	11.8	0.64	(0.34, 1.18)
No iodine irrigation	446	15	3.4	374	14	3.7	0.90	(0.46, 1.77)
Local antibiotics	9	0	0	4	0	0	NA	
No local antibiotics	624	29	4.6	574	38	6.6	0.70	(0.38, 1.29)
Cement	3	0	0	0	0	0	NA	
No cement	630	29	4.6	578	38	6.6	0.70	(0.38, 1.27)
VCM powder	6	0	0	4	0	0	NA	
No VCM powder	627	29	4.6	574	38	6.6	0.70	(0.38, 1.28)
Antimicrobial suture	627	29	4.6	572	38	6.6	0.70	(0.38, 1.27)
No antimicrobial suture	6	0	0	6	0	0	NA	
Normothermia (≥36°C)	529	23	4.3	496	30	6.0	0.72	(0.38, 1.37)
Hypothermia <36°C	85	5	5.9	69	5	7.2	0.81	(0.30, 2.18)
Unmeasured	19	1	5.3	13	3	23.1	0.23	(0.02, 2.83)

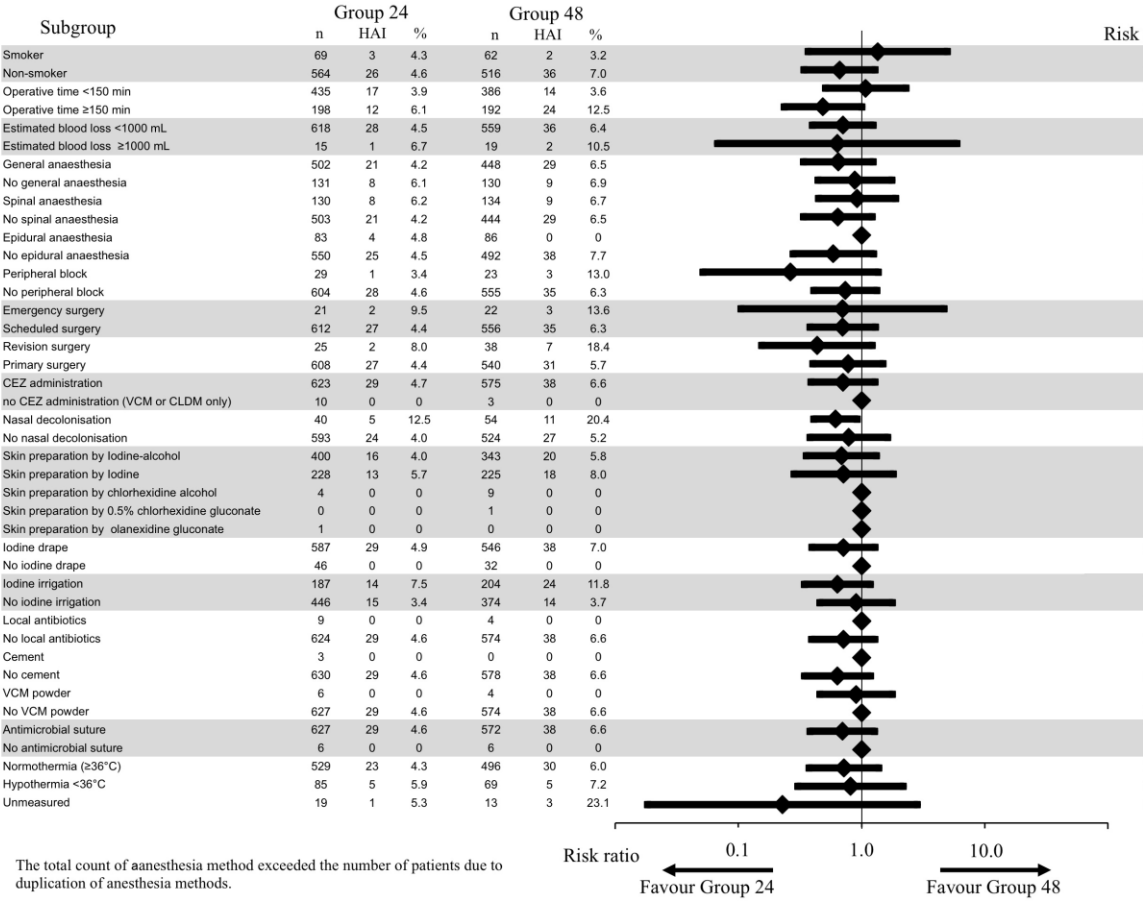

The total count of aanesthesia method exceeded the number of patients due to duplication of anesthesia methods.

© 2022 Nagata K et al. *JAMA Network Open.*

eFigure 4. Forest plot of risk ratios (95% confidence interval [CI]) for surgical site infections by subgroups (Group 24 versus Group 48)

Subgroup	Group 24 n	Group 24 SSI	Group 24 %	Group 48 n	Group 48 SSI	Group 48 %	Risk ratio	(95% CI)
Age <65 years	197	6	3.0	158	4	2.5	1.20	(0.31, 4.61)
Age 65-<80 years	265	3	1.1	258	12	4.7	0.24	(0.07, 0.90)
Age ≤80 years	171	5	2.9	162	3	1.9	1.58	(0.49, 5.05)
Sex, Male	250	6	2.4	204	10	4.9	0.49	(0.18, 1.36)
Sex, Female	383	8	2.1	374	9	2.4	0.87	(0.29, 2.56)
Diabetes	72	2	2.8	59	6	10.2	0.27	(0.06, 1.27)
No diabetes	556	12	2.2	512	13	2.5	0.85	(0.33, 2.17)
Hemodialysis	5	0	0	4	0	0	NA	
No hemodialysis	628	14	2.2	574	19	3.3	0.67	(0.30, 1.49)
Rheumatoid arthritis	27	0	0	32	1	3.1	NA	
No rheumatoid arthritis	606	14	2.3	546	19	3.5	0.66	(0.30, 1.46)
BMI <25 kg/m2	415	7	1.7	381	11	2.9	0.58	(0.19, 1.79)
BMI ≥25 kg/m2	218	7	3.2	197	8	4.1	0.79	(0.28, 2.25)
ASA 1 or 2	570	13	2.3	523	17	3.3	0.70	(0.30,1.65)
ASA 3 or 4	63	1	1.6	55	2	3.6	0.44	(0.03, 5.53)
Open reduction internal fixation	119	1	0.8	117	3	2.6	0.33	(0.04, 2.94)
Arthroplasty	273	5	1.8	245	9	3.7	0.50	(0.14, 1.78)
Arthrodesis	6	0	0	3	0	0	NA	
Osteotomy	6	0	0	2	0	0	NA	
Spine without instrumentation	108	3	2.8	98	0	0	NA	
Spine with instrumentation	94	5	5.3	93	6	6.5	0.82	(0.29, 2.36)
Arthroscopy	10	0	0	9	0	0	NA	
Bone tumor resection	0	0	0	1	0	0	NA	
Soft tissue tumor resection	3	0	0	3	0	0	NA	
Other	14	0	0	7	1	14.3	NA	
No urinary catheter	84	0	0	46	2	4.3	NA	
Urinary catheter <24 h	159	3	1.9	175	7	4.0	0.47	(0.13, 1.74)
Urinary catheter ≥24 h	390	11	2.8	357	10	2.8	1.01	(0.40, 2.53)
Surgical drain closed, <48 h	296	3	1.0	256	8	3.1	0.32	(0.07, 1.47)
Surgical drain closed, ≥48 h	258	8	3.1	253	9	3.6	0.87	(0.38, 2.02)
Surgical drain none	67	3	4.5	66	2	3.0	1.48	(0.25, 8.82)
Surgical drain not closed, <48 h	12	0	0	3	0	0	NA	
Surgical drain not closed, ≥48 h	12	0	0	3	0	0	NA	
Hospital 1	310	6	1.9	263	10	3.8	0.51	(0.15, 1.71)
Hospital 2	111	0	0	79	0	0	NA	
Hospital 3	56	2	3.6	56	3	5.4	0.67	(0.14, 3.09)
Hospital 4	48	1	2.1	66	1	1.5	1.38	(0.32, 5.93)
Hospital 5	108	5	4.6	114	5	4.4	1.06	(0.51, 2.21)
May–June 2018	219	2	0.9	157	5	3.2	0.29	(0.08, 1.04)
July–August 2018	138	6	4.3	189	6	3.2	1.37	(0.52, 3.61)
Sept-Oct 2018	137	1	0.7	140	5	3.6	0.20	(0.04, 1.06)
Nov-Dec 2018	139	5	3.6	92	3	3.3	1.10	(0.57, 2.14)

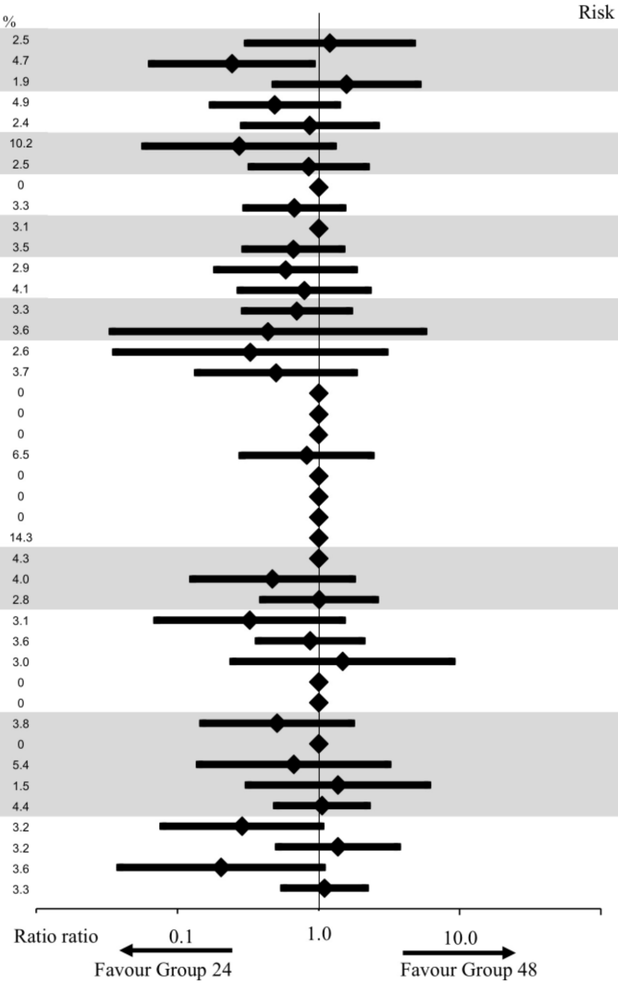

© 2022 Nagata K et al. *JAMA Network Open.*

eFigure 4. continued.

Subgroup	Group 24 n	Group 24 SSI	Group 24 %	Group 48 n	Group 48 SSI	Group 48 %	Risk ratio	(95% CI)
Smoker	69	1	1.4	62	1	1.6	0.90	(0.05, 14.94)
Non-smoker	564	13	2.3	516	18	3.5	0.66	(0.28, 1.57)
Operative time <150 min	435	6	1.4	386	6	1.6	0.89	(0.31, 2.50)
Operative time ≥150 min	198	8	4.0	192	13	6.8	0.60	(0.26, 1.37)
Estimated blood loss <1000 mL	618	13	2.1	559	18	3.2	0.65	(0.30, 1.43)
Estimated blood loss ≥1000 mL	15	1	6.7	19	1	5.3	1.27	(0.14, 11.85
General anaesthesia	502	11	2.2	448	16	3.6	0.61	(0.29, 1.32
No general anaesthesia	131	3	2.3	130	3	2.3	0.99	(0.21, 4.63
Spinal anaesthesia	130	3	2.3	134	3	2.2	1.03	(0.21, 5.02)
No spinal anaesthesia	503	11	2.2	444	16	3.6	0.61	(0.28, 1.29)
Epidural anaesthesia	83	2	2.4	86	0	0	NA	
No epidural anaesthesia	550	12	2.2	492	19	3.9	0.56	(0.22, 1.43)
Peripheral block	29	1	3.4	23	2	8.7	0.40	(0.08, 2.04)
No peripheral block	604	13	2.2	555	17	3.1	NA	
Emergency surgery	21	1	4.8	22	0	0	0.68	(0.31, 1.50)
Scheduled surgery	612	13	2.1	556	19	3.4	NA	
Revision surgery	25	0	0	38	5	13.2	1.00	(0.15, 1.22)
Primary surgery	608	14	2.3	540	14	2.6	0.89	(0.40, 1.48)
CEZ administration	623	14	2.2	575	19	3.3	0.70	(0.39, 1.28)
no CEZ administration (VCM or CLDM only)	10	0	0	3	0	0	NA	
Nasal decolonisation	40	2	5.0	54	5	9.3	0.54	(0.18, 1.61)
No nasal decolonisation	593	12	2.0	524	14	2.7	0.76	(0.31, 1.83)
Skin preparation by Iodine-alcohol	400	7	1.8	343	9	2.6	0.67	(0.20, 2.22)
Skin preparation by Iodine	228	7	3.1	225	10	4.4	0.69	(0.32, 1.50)
Skin preparation by chlorhexidine alcohol	4	0	0	9	0	0	NA	
Skin preparation by 0.5% chlorhexidine gluconate	0	0	0	1	0	0	NA	
Skin preparation by olanexidine gluconate	1	0	0	0	0	0	NA	
Iodine drape	587	14	2.4	546	19	3.5	0.69	(0.31, 1.51)
No iodine drape	46	0	0	32	0	0	NA	
Iodine irrigation	187	9	4.8	204	10	4.9	0.98	(0.51, 1.91)
No iodine irrigation	446	5	1.1	374	9	2.4	0.47	(0.19, 1.17)
Local antibiotics	9	0	0	4	0	0	NA	
No local antibiotics	624	14	2.2	574	19	3.3	0.68	(0.30, 1.51)
Cement	3	0	0	0	0	0	NA	
No cement	630	14	2.2	578	19	3.3	0.68	(0.31, 1.50)
VCM powder	6	0	0	4	0	0	NA	
No VCM powder	627	14	2.2	574	19	3.3	0.67	(0.30, 1.51)
Antimicrobial suture	627	14	2.2	572	19	3.3	0.67	(0.30, 1.49)
No antimicrobial suture	6	0	0	6	0	0	NA	
Normothermia (≥36°C)	529	12	2.3	496	15	3.0	0.75	(0.33, 1.71)
Hypothermia <36°C	85	2	2.4	69	3	4.3	0.54	(0.07, 4.27)
Unmeasured	19	0	0	13	1	7.7	NA	

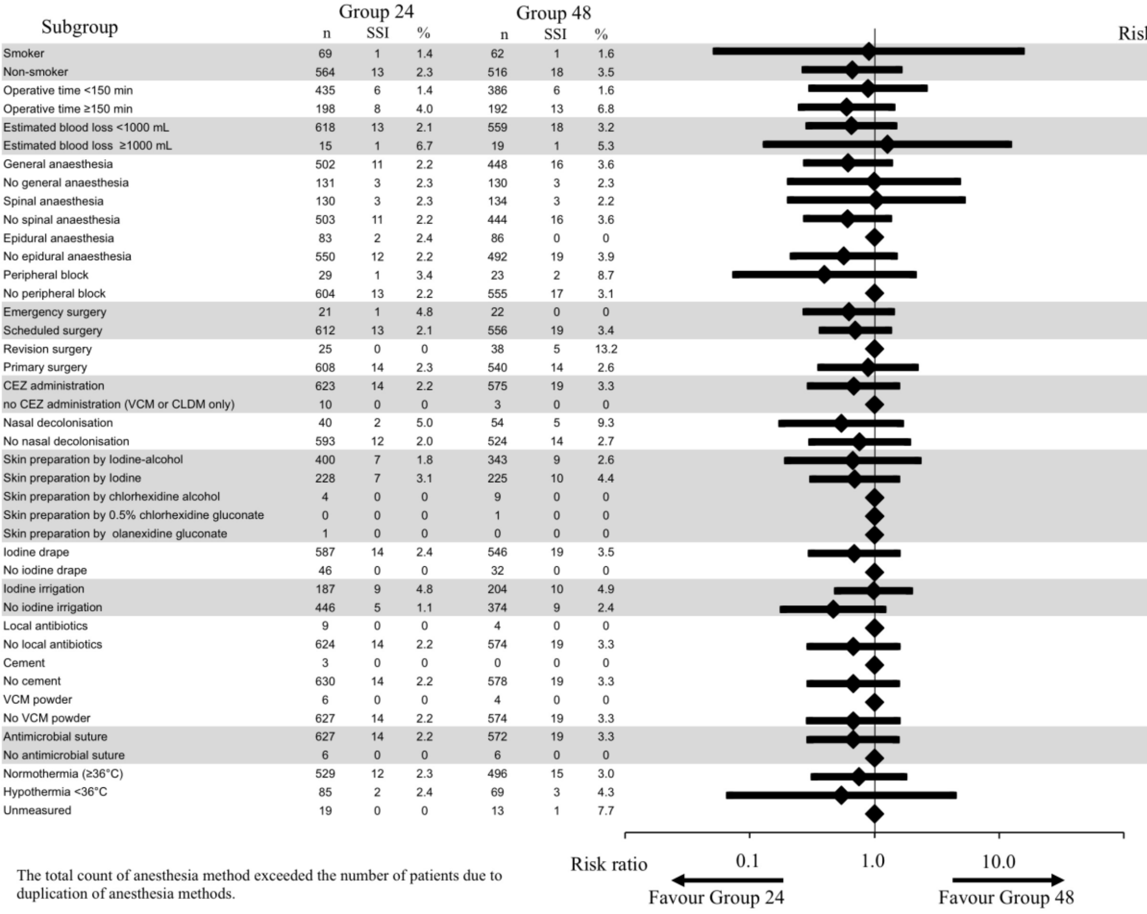

The total count of anesthesia method exceeded the number of patients due to duplication of anesthesia methods.

© 2022 Nagata K et al. *JAMA Network Open.*

eFigure 5. Probability of hospital stays in Group 24 and Group 48 during 30 days after surgery

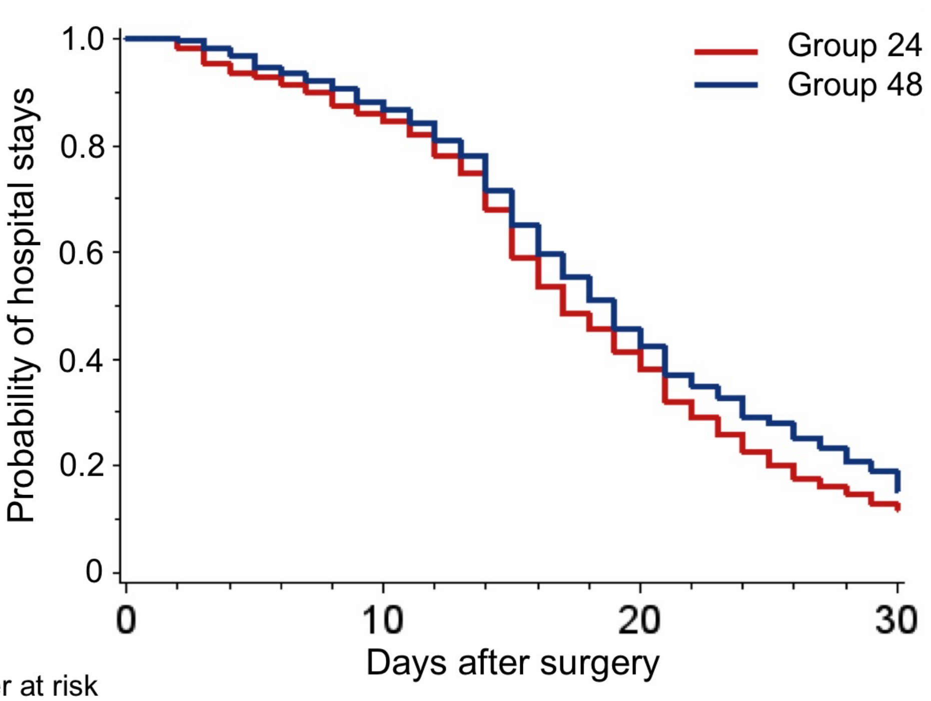

Number at risk

	Day 0	Day 10	Day 20	Day 30
Group 24	633	543	261	82
Group 48	578	510	263	110
		Hazard ratio	Cluster-robust 95% CI	
Group 24 (vs. Group 48)		1.17	(0.86 − 1.60)	

The hazard ratio of Group 24 vs. Group 48 was estimated using the Cox proportional hazard model in the intention-to-treat population for hospital stays after initial surgery to discharge. CI, confidence interval.

© 2022 Nagata K et al. *JAMA Network Open.*
