# Supplementary material for: Effect of Antimicrobial Prophylaxis Duration on Health Care–Associated Infections After Clean Orthopedic Surgery: A Cluster Randomized Trial
Source: JAMA Netw Open. 2022 Apr 12;5(4):e226095. doi: 10.1001/jamanetworkopen.2022.6095 (PMC9006110; doi:10.1001/jamanetworkopen.2022.6095)
Supplement: Supplement 3. — Nonauthor Collaborators [file jamanetwopen-e226095-s003.pdf]

Supplemental Online Content: Nonauthor Collaborators

*Indicates required information. Only first name, last name, and suffix will appear in PubMed.

*Group Name(s): OSSI Investigators							
***First Name and Middle Initial(s)**	***Last Name**	***Suffix (eg, Jr, III)**	Academic Degrees	Institution	Location (city, state/province, country)	Role or Contribution, eg, chair, principal investigator	Group (if more than 1 Group listed in the byline) and/or Subgroup (eg, Steering Committee)
Masako	Kaneko		MD	Japan Organization of Occupation	Kawasaki, Kanagawa, Ja	data collection	
Juichi	Tonosu		MD, PhD	Japan Organization of Occupation	Kawasaki, Kanagawa, Ja	data collection	
Ken	Nishimura		MD	Japan Organization of Occupation	Kawasaki, Kanagawa, Ja	data collection	
Takashi	Ishida		MD	Japan Organization of Occupation	Kawasaki, Kanagawa, Ja	data collection	
Hiroyuki	Nakarai		MD	Japan Organization of Occupation	Kawasaki, Kanagawa, Ja	data collection	
Tomoaki	Okada		MD	Japan Organization of Occupation	Kawasaki, Kanagawa, Ja	data collection	
Daisuke	Shimazu		MD	Japan Organization of Occupation	Kawasaki, Kanagawa, Ja	data collection	
Takashi	Kuniya		MD	Japan Organization of Occupation	Kawasaki, Kanagawa, Ja	data collection	
Akira	Hyodo		MD	Japan Organization of Occupation	Kawasaki, Kanagawa, Ja	data collection	
Yasuhiro	Kashima		MD	Japan Organization of Occupation	Kawasaki, Kanagawa, Ja	data collection	
Kanto	Mori		MD	Tokyo Metropolitan Tama Medica	Fuchu, Tokyo, Japan	data collection	
Keitaro	Tahara		MD	Tokyo Metropolitan Tama Medica	Fuchu, Tokyo, Japan	data collection	
Yuichi	Nagase		MD, PhD	Tokyo Metropolitan Tama Medica	Fuchu, Tokyo, Japan	data collection	
Toru	Iga		MD	Tokyo Metropolitan Tama Medica	Fuchu, Tokyo, Japan	data collection	
Euan	Morita		MD	Tokyo Metropolitan Tama Medica	Fuchu, Tokyo, Japan	data collection	
Kazuhiro	Masuda		MD	Tokyo Metropolitan Tama Medica	Fuchu, Tokyo, Japan	data collection	
Masashi	Naito		MD	Tokyo Metropolitan Tama Medica	Fuchu, Tokyo, Japan	data collection	
Yoshisato	Toriyama		MD	Tokyo Metropolitan Tama Medica	Fuchu, Tokyo, Japan	data collection	
Yusuke	Arino		MD	Tokyo Metropolitan Tama Medica	Fuchu, Tokyo, Japan	data collection	
Taihei	Urata		MD	Tokyo Metropolitan Hiroo Hospita	Tokyo, Japan	data collection	
Kazuki	Abe		MD	Tokyo Metropolitan Hiroo Hospita	Tokyo, Japan	data collection	
Shinya	Hoshikawa		MD, PhD	Tokyo Metropolitan Hiroo Hospita	Tokyo, Japan	data collection	
Takayuki	Naoe		MD	Tokyo Metropolitan Hiroo Hospita	Tokyo, Japan	data collection	
Yujiro	Hirao		MD	Tokyo Metropolitan Hiroo Hospita	Tokyo, Japan	data collection	
Takashi	Madate		MD	Tokyo Metropolitan Hiroo Hospita	Tokyo, Japan	data collection	
Hiroshi	Ito		MD	Tokyo Metropolitan Hiroo Hospita	Tokyo, Japan	data collection	
Hiroaki	Mano		MD	Tokyo Metropolitan Hiroo Hospita	Tokyo, Japan	data collection	

Supplemental Online Content: Nonauthor Collaborators

*Indicates required information. Only first name, last name, and suffix will appear in PubMed.

***First Name and Middle Initial(s)**	***Last Name**	***Suffix (eg, Jr, III)**	Academic Degrees	Institution	Location (city, state/province, country)	Role or Contribution, eg, chair, principal investigator	Group (if more than 1 Group listed in the byline) and/or Subgroup (eg, Steering Committee)
Hiroyoshi	Mastuura		MD	Tokyo Metropolitan Hiroo Hospita	Tokyo, Japan	data collection	
Yukinori	Hara		MD	Tokyo Metropolitan Hiroo Hospita	Tokyo, Japan	data collection	
Kazuhiro	Shibayama		MD	Tokyo Metropolitan Hiroo Hospita	Tokyo, Japan	data collection	
Yosei	Hirayama		MD	Tokyo Metropolitan Hiroo Hospita	Tokyo, Japan	data collection	
Shurei	Sugita		MD, PhD	Tokyo Metropolitan Cancer and In	Tokyo, Japan	data collection	
Hiroyasu	Kodama		MD	Tokyo Metropolitan Cancer and In	Tokyo, Japan	data collection	
Fujiwara	Masanori		MD	Tokyo Metropolitan Cancer and In	Tokyo, Japan	data collection	
Yoko	Ishikawa		MD	Tokyo Metropolitan Cancer and In	Tokyo, Japan	data collection	
Tatsunori	Fukui		MD	Tokyo Metropolitan Cancer and In	Tokyo, Japan	data collection	
Yukiko	Sasa		BA	Tokyo Metropolitan Geriatric Hos	Tokyo, Japan	data collection	
Shinya	Nakamura		MD	Tokyo Metropolitan Geriatric Hos	Tokyo, Japan	data collection	
Hiroshi	Hamaji		MD	Tokyo Metropolitan Geriatric Hos	Tokyo, Japan	data collection	
Akira	Kinoda		MD	Tokyo Metropolitan Geriatric Hos	Tokyo, Japan	data collection	
Yukimasa	Yamato		MD	Tokyo Metropolitan Geriatric Hos	Tokyo, Japan	data collection	
Masakazu	Kanetaka		MD	Tokyo Metropolitan Geriatric Hos	Tokyo, Japan	data collection	
Yuko	Nagai		MD, PhD	Tokyo Metropolitan Geriatric Hos	Tokyo, Japan	data collection	
