# Supplementary material for: Effect of Antimicrobial Prophylaxis Duration on Health Care–Associated Infections After Clean Orthopedic Surgery: A Cluster Randomized Trial
Source: JAMA Netw Open. 2022 Apr 12;5(4):e226095. doi: 10.1001/jamanetworkopen.2022.6095 (PMC9006110; doi:10.1001/jamanetworkopen.2022.6095)
Supplement: Supplement 4. — Data Sharing Statement [file jamanetwopen-e226095-s004.pdf]

Data Sharing Statement

Nagata K, Yamada K, Shinozaki T, et al; on behalf of the OSSI investigators. Effect of Antimicrobial Prophylaxis Duration on Health Care–Associated Infections After Clean Orthopedic Surgery. *JAMA Netw Open*. 2022;5(4):e226095. doi:10.1001/jamanetworkopen.2022.6095

Data
Data available: Yes
Data types: Deidentified participant data
How to access data: kyamadaortho-tky@umin.ac.jp
When available: With publication

Supporting Documents
Document types: Statistical/analytic code
How to access documents: kyamadaortho-tky@umin.ac.jp
When available: With publication

Additional Information
Who can access the data: researchers whose proposed use of the data has been approved
Types of analyses: for a specified purpose
Mechanisms of data availability: after approval of a proposal
